# Cost-Effectiveness of Population Level and Individual Level Interventions to Combat Non-communicable Disease in Eastern Sub-Saharan Africa and South East Asia: A WHO-CHOICE Analysis

**DOI:** 10.34172/ijhpm.2021.37

**Published:** 2021-06-07

**Authors:** Melanie Y. Bertram, Daniel Chisholm, Rory Watts, Temo Waqanivalu, Vinayak Prasad, Cherian Varghese

**Affiliations:** ^1^Department of Health Systems Governance and Financing, World Health Organization, Geneva, Switzerland.; ^2^Department of Mental Health and Substance Abuse, World Health Organization, Geneva, Switzerland.; ^3^Department of Prevention of Non-Communicable Diseases, World Health Organization, Geneva, Switzerland.; ^4^Department of Management of Non Communicable Diseases, Violence and Injury, World Health Organization, Geneva, Switzerland.

**Keywords:** Cost-Effectiveness Analysis, Economic Evaluation, Best-Buys, Non-communicable Disease, Mental Health, NCD

## Abstract

**Background:** To determine the health system costs and health-related benefits of interventions for the prevention and control of non-communicable diseases (NCDs), including mental health disorders, for the purpose of identifying the most cost-effective intervention options in support of global normative guidance on the best-buy interventions for NCDs. In addition, tools are developed to allow country contextualisation of the analyses to support local priority setting exercises.

**Methods:** This analysis follows the standard WHO-CHOICE (World Health Organization-Choosing Interventions that are Cost-Effective) approach to generalized cost-effectiveness analysis applied to two regions, Eastern sub-Saharan Africa and South-East Asia. The scope of the analysis is all NCD and mental health interventions included in WHO guidelines or guidance documents for which the health impact of the intervention is able to be identified and attributed. Costs are measured in 2010 international dollars, and benefits modelled beginning in 2010, both for a period of 100 years.

**Results:** There are many interventions for NCD prevention and management that are highly cost-effective, generating one year of healthy life for less than Int. $100. These interventions include tobacco and alcohol control policies such as taxation, voluntary and legislative actions to reduce sodium intake, mass media campaigns for reducing physical activity, and treatment options for cardiovascular disease (CVD), cervical cancer and epilepsy. In addition a number of interventions fall just outside this range, including breast cancer, depression and chronic lung disease treatment.

**Conclusion:** Interventions that represent good value for money, are technically feasible and are delivered for a low per-capita cost, are available to address the rapid rise in NCDs in low- and middle-income countries. This paper also describes a tool to support countries in developing NCD action plans.

## Background

Non-communicable diseases (NCDs) are the leading cause of disease burden globally, responsible for 58% of the global burden of disease in 2015 according to World Health Organization (WHO) estimates.^1^ Of the NCDs, the largest contributor to disease burden is cardiovascular disease (CVD), followed by cancers and mental and substance use disorders.^2^ Although excluded from direct consideration in the Millennium Development Goals, a persistent increase in the health and economic consequences due to these causes has been observed in many countries.^3^ In the post-2015 agenda, NCDs have gained prominence with global actors, with Sustainable Development Goal (SDG) 3.4 aiming to reduce by one third the risk of premature mortality (between 30 and 70 years) from NCDs and to reduce the suicide mortality rate by 2030.^4^ In addition, an important means of execution identified in goal 3A of the SDGs is to strengthen the implementation of the WHO Framework Convention on Tobacco Control (FCTC) in all countries, as appropriate.^5^ Despite being responsible for 70% of global mortality, only 1.7% of development assistance for health is directed to NCD prevention and control.^2,6^ In order for countries to achieve the SDG 3.4 goal, increased and sustained domestic resource mobilization will be required. With the growing pressure on government budgets to respond not only to health needs but also to other priorities emerging from the SDG agenda, efficient use of health resources is more relevant than ever; cost-effectiveness analyses can underpin decisions on how best to spend health resources. Further, price and tax measures which feature as potential ways of impacting health, particularly NCDs, represent a revenue stream for financing in many countries, with tobacco taxation explicitly identified in the Addis Ababa Action Agenda on financing for development.^7^

Action on NCDs is increasing at the country level, and while 86% of WHO Member States now report that they have an NCD action plan, only 53% have multisectoral, integrated plans.^8^ In many low- and middle-income countries progress is being held back by a number of factors: absence of strong political will, prioritizing trade and commercial interests over public health policy, paucity of expertise in NCD policy dialogue, and lack of capacity and resources to respond to demands for technical assistance. WHO continually strives to provide tools and technical assistance to support Member States in the development of Action Plans for NCDs, and one of those tools is international guidance on the relative cost-effectiveness of different prevention and treatment interventions. The analysis presented here underpins the 2017 update of Appendix 3 of the WHO’s Global Action Plan for NCDs 2013-2020. This appendix is essentially a menu of policy options and cost-effective interventions for the prevention and control of major NCDs, to assist Member States in implementing, as appropriate, for national context (without prejudice to the sovereign rights of nations to determine taxation among other policies), actions to achieve the nine voluntary global targets. The 2017 Appendix was considered by the World Health Assembly in May 2017, and passed as resolution A70/27 enshrining a set of so-called best buy interventions in the global NCD policy agenda.

The prevention and management of mental and behavioural disorders is all too often omitted from NCD planning, advocacy and system strengthening. This is despite similarities in the underlying risk factors for, and chronic consequences of these conditions, as well as substantial evidence of co-morbidities between mental disorders and other major NCDs.^9^ Moreover, and as reflected in WHO treatment guidelines for mental disorders and other NCDs (the mhGAP Intervention Guide and the Package of Essential Non-Communicable Disease interventions [PEN package], respectively), the underlying principles and professional competencies required for effective management of these disorders in non-specialised healthcare settings are very similar.^10,11^ Accordingly, we present an overview of the cost-effectiveness of NCD and MNS (mental, neurological and substance use disorders) policy options. In line with the WHO Global Action Plan for the Prevention and Control of NCDs 2013-2020, the analysis focusses on the four diseases and four risk factors which comprise the majority of the NCD burden^12^ along with the major drivers of mental health burden as identified through the mhGAP package.

This article is part of an update of the WHO-Choosing Interventions that are Cost-Effective (CHOICE) programme of work.^13^ The CHOICE approach to cost-effectiveness is unique in three ways. Firstly, generalized cost-effectiveness is used, enabling critical analysis of the current package of available interventions, which enables us to view the allocative efficiency of current care. Secondly, all currently recommended interventions with adequate evidence are included in the analysis, initially individually and then as packages of care based on combining the most cost-effective interventions. Finally, common methodology and assumptions are used across different disease areas, enabling interventions for different diseases to be compared.

A number of cost-effectiveness analyses for NCDs and MNS disorders were undertaken by WHO-CHOICE in a series published in 2012, using a baseline year of 2005.^14-16^ The analyses concluded that there are a number of interventions that are very cost-effective, including demand reduction strategies for tobacco and alcohol, multi-drug therapy for primary and secondary prevention of CVD, epilepsy treatment and some cancer control options. Interventions for chronic lung diseases and schizophrenia were generally considered to have less favourable cost-effectiveness ratios due to the chronic nature of the conditions and more modest effect sizes for assessed interventions.

 The current study represents the first thorough re-analysis of the cost-effectiveness of interventions for NCDs and MNS disorders by WHO since the 2012 publication. The specific aim of this analysis is to identify any major changes in the cost-effectiveness of NCD and MNS interventions, and to reassess the validity of the best-buys for NCDs and MNS disorders. The analysis platform has been updated, new health impact models developed and a broader range of preventive strategies have been included in the analysis; for example, new WHO recommendations for preventive food policies and physical inactivity have been included for the first time in WHO-CHOICE analyses.

## Key Messages

**Implications for policy makers**

- This paper is a complete update of the original WHO-CHOICE (World Health Organization-Choosing Interventions that are Cost-Effective ) analysis, now with 2010 as a baseline year, with an updated list of interventions included which aligns with current WHO Guidelines and other guidance documents in this field.
- This analysis underlies the Appendix 3 of the Global Action Plan for non-communicable diseases (NCDs) which was supported by the WHO Member States in the World Health Assembly in 2017.
- This is a first attempt to bring all of these diseases and risk factors together in a single publication, to support the development of an essential package of health services, combined for NCDs and mental health disorders. We focus on services delivered across two health service platforms: population level services and primary clinical services. This is important as countries with weak health systems that are not in a position to scale up clinical services immediately can opt to implement an evidence-based, cost-effective set of population health services as the first step on the path to universal health coverage.
- Cost-effective interventions exist for NCDs and mental health disorders, providing evidence that interventions for these conditions should be fully integrated into universal health coverage benefit packages.

**Implications for public**

This analysis confirms that cost-effective and affordable interventions exist for the biggest drivers of the non-communicable disease (NCD) burden. Many of these interventions focus on prevention, such as tobacco cessation measures, however good value-for-money treatment options are available for all disease areas. This means that there are interventions that satisfy often used criteria for addition of interventions to publicly financed health benefit packages. Making these interventions accessible to the public at a price they can afford is the way for countries to progress towards universal health coverage. The results presented in this paper could be useful to support patient groups in motivating for their interventions to be added to health benefit packages and financed by national insurance mechanisms.

## Methods

The methods of the WHO-CHOICE project have been published in full previously,^17^ and mental health and NCDs specific methods were first published in 2005 and 2012, respectively. In addition, an accompanying methods paper within this series describes the generic WHO-CHOICE methods in detail.^18^ In this paper we describe specific methodology related to updating the analytical work for NCDs and MNS disorders, including brief overviews of the models developed and the intervention assumptions used, as well as additional detail for new impact models developed for sodium reduction, trans fat elimination and physical activity promotion. The analysis uses epidemiological and cost data for 2010, for the Eastern sub-Saharan Africa and South-East Asia Global Burden of Disease regions. Countries included in these regions are presented in the accompanying methods paper.

In brief, the WHO-CHOICE project evaluates interventions across a range of diseases and risk factors, using a common methodology to allow for comparison and integration of results from single diseases into a sector-wide analysis. Health outcomes are measured using the OneHealth/Spectrum suite of impact models. The OneHealth Tool is a software tool designed to inform national strategic health planning in low- and middle-income countries by developing cost and health impact projections. It is freely available for download, along with supporting documentation and all methodological inputs from https://www.avenirhealth.org. Health outcomes are reported as the gain in healthy life years (HLYs) due to a specific intervention. The use of HLY enables comparison across different diseases, allowing for priority setting across the health sector. Disease weights used in the calculation of HLYs are from the Global Burden of Disease study, 2010.^19^ Costs are measured from the perspective of the health system. The OneHealth tool is used to assess patient level intervention delivery costs, and programme costs are added to this using a standardized methodology.^20^ The full cost of delivering the intervention, including all pharmaceuticals and tests, is calculated.^21^ Costs incurred outside of the health system by patients, for example to travel to a health facility, are not included in the analysis.^13^ All costs and impacts are assessed over a 100 year time frame, an update on previous WHO-CHOICE methodology.^18^ All costs are discounted at 3% per annum, and HLY are presented both undiscounted and with a 3% per annum discount rate. Results are presented as average cost-effectiveness ratios (ACERs), and through the development of an expansion path showing the allocatively efficient package of interventions for NCD and MNS disorders. Due to the nature of generalised cost-effectiveness analysis, no decision rule is used to indicate if an intervention is cost-effective or not, rather the expansion path is presented and a country would select interventions until the budget is exhausted. An expansion path is calculated by identifying the most cost-effective intervention from the list of ACERs, following which we calculate the incremental cost-effectiveness ratio of adding the next most cost-effective intervention to the package. This can be either a new intervention, or an increase in coverage of the same intervention. This involves calculation of the incremental health benefits – which are in effect lower than implementing an individual intervention, as some health benefit has already been obtained with the first intervention – and incremental costs which should include economies of scale thus not be entirely additive between interventions. Interventions can be dominated, meaning they have lower health benefit and higher costs, at each incremental addition to the expansion path. The final result is a step-wise progression through the most cost-effective package of services that could be provided.

For analysis of NCDs and MNS disorders, the previously published disease impact models^14-16,22,23^ were all re-programmed in the Spectrum platform, with disease epidemiology updated to 2010 based on the Global Burden of Disease analysis for that year.^24^ Similarly, disability weights for health states were drawn from the Global Burden of Disease analysis of the same year.^19^ Models have been developed for CVD (based on the absolute risk approach), diabetes, asthma, chronic lung disease, breast cancer, cervical cancer and colorectal cancer. Overarching the disease models is a set of risk factor models for tobacco, alcohol, physical inactivity and unhealthy diet (specifically focussed on sodium and trans fats). For MNS disorders, cost-effectiveness models have been developed and implemented for depression, anxiety, psychosis, bipolar disorder, epilepsy, and hazardous and harmful alcohol use. Again, previously published modelling methodologies^15^ were transferred to the Spectrum platform, using updated disease epidemiology drawing from the global burden of disease studies. All models are publicly available through the Spectrum platform (https://www.avenirhealth.org), with full details of all modelling methods and data inputs available in the user manuals.

This study evaluates 77 interventions for prevention and control of NCDs and MNS disorders. The majority of interventions have been evaluated at three coverage levels, 50%, 80% and 95%. This enables evaluation of the (dis)economies of scale associated with increased coverage. For legislative interventions, the interventions have been evaluated at the highest intensity of implementation where this is relevant, for example for the MPOWER Tobacco reduction policies, or at full implementation. Interventions draw on WHO guidelines in the first instance, and other guidance documents where these are not available. Specifically, for CVD, diabetes, asthma and chronic obstructive pulmonary disease (COPD), the PEN Package is evaluated.^11^ For cancer, treatment guidelines produced in 2014 are evaluated.^25^ For tobacco we draw on the MPOWER measures^26^ that are consistent with the key demand reduction provisions of the WHO FCTC.^27^ For dietary and nutrition policies, we reference the SHAKE package^28^ of sodium reduction policy options, and the technical report of the Fiscal Policies for Diet expert consultation meeting convened by WHO for sugar and sweetened beverages.^29^ For physical activity we use the draft technical package containing evidence of which policy interventions work.^30^ For MNS disorders, we draw from the mhGAP Intervention Guide and the Global Strategy for reducing the harmful use of alcohol.^10^

In the first instance all interventions are individually compared to the “null,” a hypothetical scenario in which the effects of all currently implemented interventions are removed. One exception to this is asthma treatment, which is specifically recommended as a stepped approach to asthma treatment and control, thus packages of care are evaluated. Subsequently, the marginal addition of interventions is evaluated in order to develop an essential package of efficient care for addressing NCDs and mental disorders. Supplementary file 1 provides information on full intervention descriptions and impact sizes used in the cost-effectiveness analysis.

###  Intervention Costs

Costing of interventions follows a standardized framework developed for WHO-CHOICE, and includes patient level delivery costs, programmatic costs and health system costs. Interventions are costed assuming health system capacity is available to support the intervention. Quantity assumptions are based on adherence to WHO guidelines for the intervention of interest. Prices come from the WHO-CHOICE price database.^18^ Programme costing is done in accordance with the methodology outlined in the WHO-CHOICE programme costing paper.^20^ Costs are discounted at 3% per annum, and capital expenses are annuitized over the lifetime of the good. All prices are in 2010 International Dollars. 2010 was chosen as the baseline year in line with the 2010 Global Burden of Disease study epidemiological data which forms the base of many of the disease models used in WHO-CHOICE. Supplementary files 2, 3 and 4 provide costing assumptions for each of the interventions included in the analysis.

###  Sensitivity Analysis

 One-way sensitivity analysis was performed on key input parameters to estimate the certainty of the results. We varied the discount rate on health impacts, using 0% and 3%, three coverage rates are used (50%, 80% and 95%) and the price of non-traded inputs was varied by plus and minus 25%. Due to the number of input parameters, and the limits of our analytic platform, we are unable to perform probabilistic sensitivity analysis.

## Results

 ACERs for NCD and mental health treatment and prevention interventions vary widely, but there are interventions which represent very good value for money and which could form a health benefit package for NCDs in low-income settings, with additional options for countries transitioning toward universal health coverage. We do not use any decision rule or threshold in defining “good value for money” but rather consider relative ACER values within this package of interventions.

**Figure 1:**
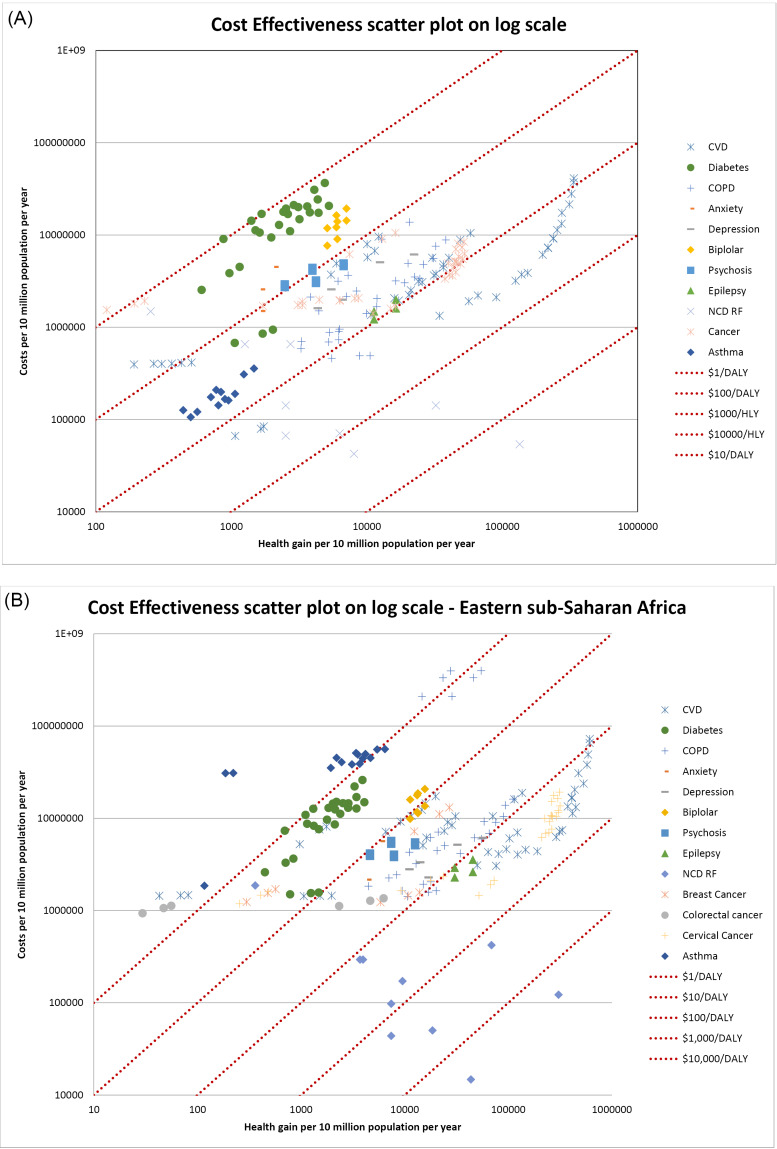
Log-Log Graphs of Costs and Benefits, Representing the ACER Values, of Each Intervention in (A) South-East Asia and (B) Eastern Sub-Saharan Africa.Diagonal lines represent order of magnitude changes in ACER, beginning at <$1/HLY, $1-10/HLY, $10-100/HLY, $100-1000/HLY, $1000-$10000/HLY. Abbreviations: HLY, healthy life year; ACER, average cost-effectiveness ratio; CVD, cardiovascular disease; COPD, chronic obstructive pulmonary disease; NCD, non-communicable disease; DALY, disability-adjusted life year.

ACERs are presented for each individual intervention in Supplementary file 5. Cost-effectiveness varies by orders of magnitude across the group of interventions (Figures 1A and 1B). In Eastern sub-Saharan Africa, more than 60 policy options are available which cost less than Int. $100 per HLY gained, with an additional 70 plus policy options under Int. $1000 per HLY gained (Note that some interventions may be repeated at different coverage levels in the analysis). However at the opposite end of the scale, comprehensive cancer palliation programmes have the highest cost-effectiveness ratio of around Int. $30000 per HLY gained. Similarly in South East Asia, 29 policy options cost less than Int. $100 per HLY and an additional 108 are available for under Int. $1000 per HLY gained. As with Eastern sub-Saharan Africa, comprehensive cancer care was the highest cost per HLY of the interventions evaluated. The cost-effectiveness ratio of each individual policy option is shown graphically on a log-log scale (Figures 1A and 1B).

**Figure 2:**
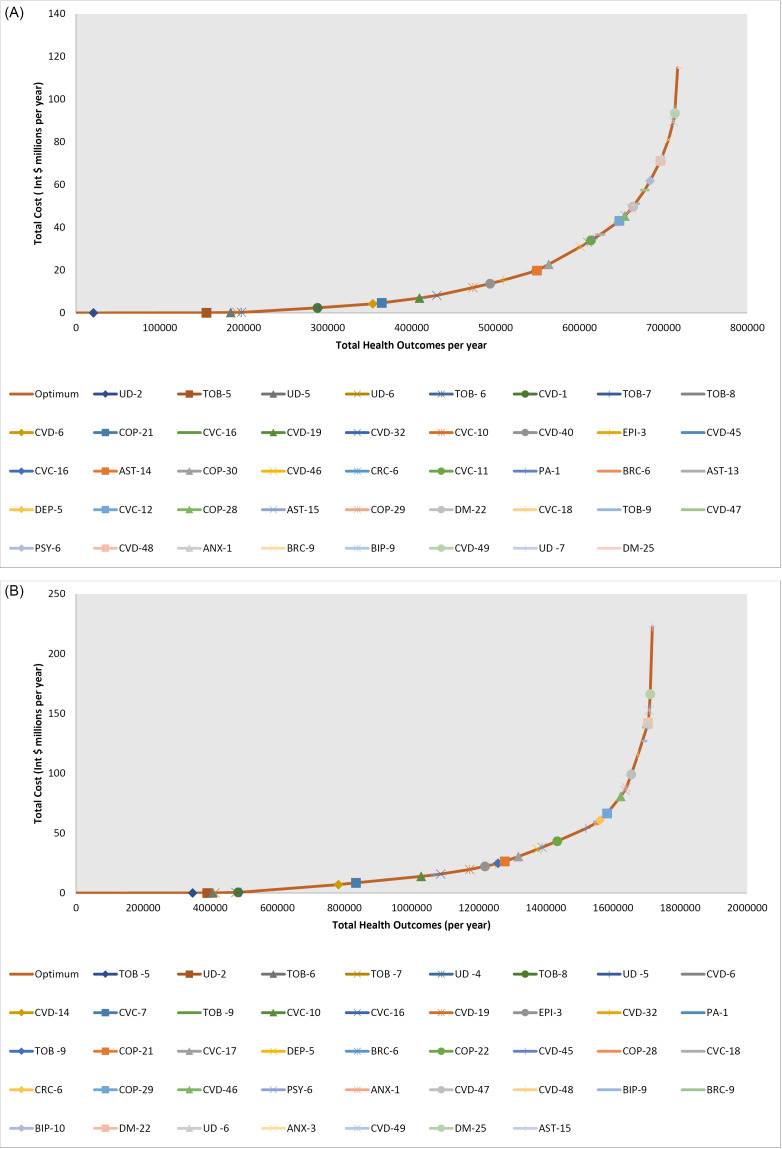
Expansion Paths Showing the Most Allocatively Efficient Mix of Health Intervention That Could Be Added Sequentially to a Health Benefit Package in (A) the Eastern Sub-Saharan African and (B) the South East Asia Region. Abbreviations: TOB, tobacco; UD, unhealthy diet; CVD, cardiovascular disease; COP, chronic obstructive pulmonary disease; CVC, cervical cancer; EPI, epilepsy; AST, asthma; DEP, depression; BRC, breast cancer; PSY, psychosis; CRC, colorectal cancer; BIP, bipolar disorder; ANX, anxiety disorder; DM, diabetes mellitus.

Packages of care, or expansion paths, for NCDs and MNS disorders can be developed at different price points to respond to a country’s ability to expand their health budget to begin scaling up action in these areas (Table, Figure 2A and 2B). For less than Int. $1 per capita, countries can begin implementation of population level prevention interventions for tobacco and unhealthy diets, preventive pharmaceutical therapy for CVD in those at high risk and vaccination against human papillomavirus. In the South East Asia region (Figure 2A), additional interventions for COPD treatment and expansion of preventive CVD therapy in a lower risk group could also be added for the cumulative total of Int. $1 per capita. At a total cost of Int. $5 per capita, we begin to see treatment interventions for epilepsy, depression and asthma. As countries move toward progressive realisation of universal health coverage, they can continue to expand the NCD and MNS package of care to cover higher level treatment interventions, such as cancer care and tertiary care for NCDs.

**Table:** Expansion Path Numbers

**Eastern Sub-Saharan Africa Expansion Path**				**South East Asia Expansion Path**			
**Intervention Name**	** Pathway Cost Per Year **	** Pathway Benefit Per Year **	** ICER**	**Intervention Name**	** Pathway Cost Per Year **	**Pathway Benefit Per Year **	**ICER **
**Origin**	** $- **	** - **	** $- **	**Origin**	** $- **	** - **	** $- **
TOB-5	$49082	347576	$0.14	UD-2	$4710	20771	$0.23
UD-2	$63829	391166	$0.34	TOB-5	$58710	155371	$0.40
TOB- 6	$114047	407877	$3.01	UD-5	$200714	184490	$4.88
TOB-7	$157897	414547	$6.57	UD-6	$243180	191730	$5.87
UD-5	$580408	476559	$6.81	TOB- 6	$314049	197416	$12.46
TOB-8	$678332	483229	$14.68	CVD-1	$2966346	287806	$29.34
UD-6	$850281	491825	$20.00	TOB-7	$3033131	290075	$29.43
CVD-14	$8023496	782079	$24.71	CVD-6	$5245123	353839	$34.69
COP-27	$8748115	810590	$25.42	CVD-19	$8035728	398827	$62.03
CVC-10	$16388055	1057082	$30.99	TOB-8	$8177732	401096	$62.59
CVD-6	$17756430	1098129	$33.34	CVD-32	$9573510	419583	$75.50
CVD-19	$22161917	1184333	$51.11	COP-21	$10382562	430153	$76.54
EPI-3	$24808645	1229781	$58.24	EPI-3	$12025297	446599	$99.88
AST-15	$25224568	1236230	$64.49	CVC-10	$16321185	489449	$100.26
CVD-32	$27428061	1267779	$69.84	CVD-43	$21813334	526159	$149.61
CVC-11	$31223964	1312502	$84.88	CVD-45	$25322408	546687	$170.94
TOB-9	$31517860	1315834	$88.19	AST-14	$25495547	547642	$181.47
DEP-5	$37864837	1372782	$111.45	CVC-11	$26968766	554064	$229.38
CVD-45	$52513473	1458046	$171.80	DEP-5	$33576991	576414	$295.67
COP-28	$63968520	1519490	$186.43	CVD-46	$44809142	614236	$296.97
BRC-6	$67459505	1533387	$251.21	COP-22	$48469165	626297	$303.46
CVC-17	$70919287	1546382	$266.23	COP-28	$51110328	634754	$312.29
COP-29	$77512757	1567494	$312.32	AST-15	$51317108	635271	$400.30
PSY-6	$82697939	1580090	$411.65	CVC-12	$52050375	637091	$402.86
CVD-46	$100519497	1620509	$440.92	CRC-6	$55823480	645795	$433.48
CRC-6	$103420361	1626773	$463.12	BRC-6	$59697157	653957	$474.60
BRC-9	$113027607	1639852	$734.52	DM-22	$60789496	655986	$538.32
CVD-48	$139965630	1674354	$780.76	COP-29	$64941097	663285	$568.84
BIP-9	$151364515	1687778	$849.19	TOB-9	$65596978	664418	$578.62
ANX-2	$155633047	1692362	$931.03	PSY-6	$70386040	671169	$709.45
BIP-10	$157814751	1694600	$975.19	CVD-48	$87336890	694893	$714.48
CVC-18	$169605395	1705932	$1040.45	BRC-9	$94072236	703009	$829.90
DM-22	$171300568	1707413	$1144.39	CVC-18	$96227224	704543	$1,405.14
UD -7	$173169339	1707774	$5175.56	ANX-1	$98698307	706206	$1,485.57
DM-25	$187593253	1710377	$5541.32	BIP-9	$107737131	712278	$1,488.67
CVD-49	$208191336	1712136	$11714.10	UD -7	$109222543	712532	$5,848.65
				DM-25	$132102578	715764	$7,078.03
				CVD-49	$148827020	717500	$9,635.34

Abbreviations: ICER, incremental cost-effectiveness ratio; TOB, tobacco; UD, unhealthy diet; CVD, cardiovascular disease; COP, chronic obstructive pulmonary disease; CVC, cervical cancer; EPI, epilepsy; AST, asthma; DEP, depression; BRC, breast cancer; PSY, psychosis; CRC, colorectal cancer; BIP, bipolar disorder; ANX, anxiety disorder; DM, diabetes mellitus.

 Sensitivity analysis indicated that the order of magnitude of cost-effectiveness ratios and rank ordering of interventions is relatively fixed even in a situation where the values of input parameters vary. For changes in discount rates, coverage and commodity costs, the rank ordering of interventions by ACER and the ordering of additions to the expansion path remained unchanged. Interventions that rely largely on non-traded inputs are highly cost-effective and varying the price does not influence the order of magnitude of the cost-effectiveness ratio. Similarly, for clinical interventions pharmaceuticals are the dominant cost input. In order to improve cost-effectiveness ratios, and potentially increase affordability, the price of pharmaceutical inputs would need to be reduced. Cost-effectiveness ratios largely improve as coverage improves due to economies of scale being present within the overarching programme support costs.

## Discussion

 More than a decade after an initial global analysis of the cost-effectiveness of interventions for NCD and MNS disorders, this study sought to re-examine and update these CHOICE estimates based on latest treatment guidelines, WHO policy recommendations, epidemiology and prices. Findings show that little has altered in terms of the general conclusions, namely that population level interventions for prevention of NCDs remain some of the most cost-effective interventions available, however there are some treatment options which continue to provide excellent value for money regardless of the setting.

 There are a number of limitations to this analysis that should be noted. The estimates are presented at the Regional level, and based on global, normative guidelines. Global estimates of impact and service delivery models are used, which may not be easily transferrable to all settings so should be interpreted with caution. This means that they do not specifically represent any single country and therefore should be appropriately contextualised before being considered in country level policy development. The calculation platform in Spectrum does not allow us to perform probabilistic uncertainty analysis. We have undertaken univariate sensitivity analysis which confirm the order of magnitude of estimates, we would therefore recommend caution in the over-interpretation of point estimate results.

 Global discourse around the NCD agenda indicates that scaling up NCD plans will be reliant on the possibility to increase the availability of domestic resources for these policy options. This analysis supports the notion that for very minimal increases in spending, there is potential for countries to start addressing the unmet need of NCD prevention and treatment. Particularly in low resource settings, making these investments now in preventive services can mitigate some of the burden of future healthcare costs anticipated as the epidemiological transition continues.

Additionally, this analysis shows that there are highly cost-effective prevention and treatment options – at less than $100 per HLY gained – which are considered “best buys” within the Appendix 3 of the Global Action Plan for Non-Communicable Disease.^31^ In most cases, prevention and early detection programmes are relatively more cost-effective than the often costly treatment programmes, and should be rapidly implemented in all settings to prevent large future treatment costs. Moreover the list of cost-effective interventions could be useful when prioritizing options within the context of limited resources in country. Although the sole focus of results presented in this paper, cost-effectiveness analysis is only one part of the priority setting process, not a single criterion, and needs to be considered along with other concerns like equity, gender and human rights, and the need to avoid financial impoverishment on the part of those who seek care. These other criteria have not been considered within this global paper, but should be simultaneously considered at the country level.

 When looking at the list of ACER values and their rank ordering, in most cases the more cost-effective interventions are fiscal and regulatory measures, which have a low per-capita cost, and if well implemented and enforced can have a large population level health impact. These interventions do not rely on a well-functioning health system and are not human resource intensive. Only health system costs are included in line with the WHO-CHOICE methodology, whereas the use of a societal perspective could change these ACER values. Whilst some clinical services, such as pharmaceutical therapy for preventing CVD or treatment of mild depression, do have low ACER values, in general as the complexity of the intervention increases, so too does the ACER value indicating it is relatively less cost-effective. In some instances ACER values could be lowered as pharmaceutical prices are reduced as they come off patent or other pricing tools are used to negotiate lower costs.

 It is important to be mindful of that these are global normative estimates of cost-effectiveness. This means that they are based on delivery of services based on global guidelines and best practice, and therefore do not represent the current contextual cost-effectiveness in any particular setting, but they do provide indicative information on the likely cost-effectiveness in an average country in the region. To support country contextualisation of this type of analysis, WHO-CHOICE has developed a generalised cost-effectiveness analysis tool which countries can use to support the data needs of their NCD action plan development.

The cost-effectiveness analysis for treatment is performed based on delivery using WHO clinical practice guidelines, and assuming a functioning health system with the capacity to deliver these interventions. At present, many countries will need to make significant investments in health systems capacity to support the implementation of many of the clinical intervention options. Health system investments are shared across all the vertical disease programmes, and the OneHealth tool can be used to assess capacity constraints in the short term which need to be addressed to enable NCD and MNS interventions to be scaled up. This does not, however, mean that NCD and MNS interventions should be ignored until such capacity is available, with population level interventions available which do not rely on health systems capacity.^32^

 WHO’s experience in providing technical assistance to countries in support of the implementation of the FCTC’s identified demand reduction strategies demonstrates that reducing the prevalence of tobacco use, particularly through taxation, can prevent millions of tobacco-attributable deaths globally and contribute to the achievement of global health objectives. Leveraging the success of the tobacco control community to support the implementation of other highly cost-effective NCD interventions as identified in this paper and politically supported by the Member States of WHO puts countries squarely in the drivers seat on the road to universal health coverage.

## Acknowledgements

 The authors wish to acknowledge the contribution of Nintsoa Ralaidovy and Jeremy Lauer from the Department of Health Systems Governance and Financing at WHO for contributing the Cancer results included in this analysis, Andre Ilbawe, Gojka Roglic, Nils Billo and Oyere Onuma from the Department of Non Communicable Diseases, Violence and Injury at WHO for reviewing technical data on treatment interventions, Fiona Bull, Chizuru Nishida, Leendert Nederveen, Benn McGrady, Mark Goodchild, Anne-Marie Perucic and Jeremias Paul Jr from the Department of Prevention for Non Communicable Diseases for reviewing technical data on risk factor interventions, and Daniela Moro for technical assistance with intervention modelling of mental health interventions.

## Ethical issues

 No ethical approval was sought as this is a secondary data analysis.

## Competing interests

 Authors declare that they have no competing interests.

## Authors’ contributions

 MYB was overall responsible for the development and population of cost and impact models for diabetes, CVD, asthma, COPD, tobacco, physical inactivity and sodium reduction. She undertook the overall data analysis and interpretation and wrote the manuscript. DC was responsible for the development and population of cost and impact models for depression, anxiety, psychosis, bipolar disorder, epilepsy, and hazardous and harmful alcohol use and interpretation of the results of these models. RW generated data on cost, impact and cost-effectiveness for each intervention using these models including quality control and technical input to development, as well as assisting in overall analysis and interpretation. TW provided technical input on salt, transfat and physical activity interventions. VP provided technical input on tobacco interventions. CV provided technical input on management interventions. All authors have contributed to the final manuscript.

## Disclaimer

 MYB, DC, RW, TW, VP and CV are staff members of the WHO. The views expressed in this paper are solely the responsibility of the named authors and do not necessarily reflect the decisions or stated policy of the WHO or its Member States.

## Authors’ affiliations

^1^Department of Health Systems Governance and Financing, World Health Organization, Geneva, Switzerland. ^2^Department of Mental Health and Substance Abuse, World Health Organization, Geneva, Switzerland. ^3^Department of Prevention of Non-Communicable Diseases, World Health Organization, Geneva, Switzerland. ^4^Department of Management of Non Communicable Diseases, Violence and Injury, World Health Organization, Geneva, Switzerland.

## Supplementary files


Supplementary file 1. Intervention Descriptions and Impact Sizes.

Supplementary file 2. Costing Inputs for NCD Interventions.

Supplementary file 3. Costing Inputs for Risk Factor Interventions.

Supplementary file 4. Costing Inputs for MNS Interventions.

Supplementary file 5. Cost, Impact and ACER Values for All Interventions.
