# Supplementary material for: Cost-Effectiveness of Population Level and Individual Level Interventions to Combat Non-communicable Disease in Eastern Sub-Saharan Africa and South East Asia: A WHO-CHOICE Analysis
Source: Int J Health Policy Manag. 2021 Jun 7;10(11):724–33. doi: 10.34172/ijhpm.2021.37 (PMC9278376; doi:10.34172/ijhpm.2021.37)
Supplement: Supplementary file 1 — Intervention Descriptions and Impact Sizes. [file ijhpm-10-724-s001.pdf]

Article title: Cost-Effectiveness of Population Level and Individual Level Interventions to Combat Non-communicable Disease in Eastern Sub-Saharan Africa and South East Asia: A WHO-CHOICE Analysis

Journal name: International Journal of Health Policy and Management (IJHPM)

Authors' information: Melanie Y. Bertram[1]*, Daniel Chisholm[2], Rory Watts[1], Temo Waqanivalu[3], Vinayak Prasad[3], Cherian Varghese[4]

[1]Department of Health Systems Governance and Financing, World Health Organization, Geneva, Switzerland.

[2]Department of Mental Health and Substance Abuse, World Health Organization, Geneva, Switzerland.

[3]Department of Prevention of Non-Communicable Diseases, World Health Organization, Geneva, Switzerland.

[4]Department of Management of Non Communicable Diseases, Violence and Injury, World Health Organization, Geneva, Switzerland.

(*Corresponding author: bertramm@who.int)

Supplementary file 1. Intervention Descriptions and Impact Sizes

Number	Cardiovascular Disease	Intervention description	Impact size	Impact reference
1	Combination therapy for prevention of CVD in those with 30%+ risk	Drug therapy (including glycaemic control for diabetes mellitus and control of hypertension and cholesterol using a total risk approach) to persons with high risk (≥ 30%) of a fatal and non-fatal cardiovascular event in the next 10 years	1.05 mmol/L change in cholesterol<br>5.9mmHg reduction in systolic blood pressure	[1] Taylor F, et al Statins for the primary prevention of cardiovascular disease. Cochrane Database of Systematic Reviews 2013, Issue 1.<br>[2] Law et al. Use of blood pressure lowering drugs in the prevention of cardiovascular disease: meta-analysis of 147 randomised trials in the context of expectations from prospective epidemiological studies. BMJ 2009;338:b1665
2	Blood pressure lowering treatment for those with SBP > 160 mmHG and total CVD risk < 30%	Blood pressure lowering drug therapy for those who do not qualify for preventive measures based on the absolute risk approach but have a systolic blood pressure of greater than 160mmHG	5.9mmHg reduction in systolic blood pressure	[2] Law et al. Use of blood pressure lowering drugs in the prevention of cardiovascular disease: meta-analysis of 147 randomised trials in the context of expectations from prospective epidemiological studies. BMJ 2009;338:b1665
3	Blood pressure lowering treatment for those with SBP > 140 mmHG and total CVD risk < 30%	Blood pressure lowering drug therapy for those who do not qualify for preventive measures based on the absolute risk approach but have a systolic blood pressure of greater than 140mmHG	5.9mmHg reduction in systolic blood pressure	[2] Law et al. Use of blood pressure lowering drugs in the prevention of cardiovascular disease: meta-analysis of 147 randomised trials in the context of expectations from prospective epidemiological studies. BMJ 2009;338:b1665
4	Cholesterol lowering treatment for those with Chol > 8 mmol/L and total CVD risk < 30%	Cholesterol lowering drug therapy for those who do not qualify for preventive measures based on the absolute risk approach but have total blood cholesterol of greater than 8 mmol/L	1.05 mmol/L change in cholesterol	[1] Taylor F, et al Statins for the primary prevention of cardiovascular disease. Cochrane Database of Systematic Reviews 2013, Issue 1.
5	Cholesterol lowering treatment for those with Chol > 6 mmol/L and total CVD risk < 30%	Cholesterol lowering drug therapy for those who do not qualify for preventive measures based on the absolute risk approach but have	1.05 mmol/L change in cholesterol	[1] Taylor F, et al Statins for the primary prevention of cardiovascular disease. Cochrane Database of Systematic Reviews 2013, Issue 1.

		total blood cholesterol of greater than 6 mmol/L		
6	Combination therapy for prevention of CVD in those with 20%+ risk	Drug therapy (including glycaemic control for diabetes mellitus and control of hypertension and cholesterol using a total risk approach) to persons with high risk (≥ 20%) of a fatal and non-fatal cardiovascular event in the next 10 years	1.05 mmol/L change in cholesterol<br>5.9mmHg reduction in systolic blood pressure	[1] Taylor F, et al Statins for the primary prevention of cardiovascular disease. Cochrane Database of Systematic Reviews 2013, Issue 1.<br>[2] Law et al. Use of blood pressure lowering drugs in the prevention of cardiovascular disease: meta-analysis of 147 randomised trials in the context of expectations from prospective epidemiological studies. BMJ 2009;338:b1665
7	Blood pressure lowering treatment for those with SBP > 160 mmHG and total CVD risk < 20%	Blood pressure lowering drug therapy for those who do not qualify for preventive measures based on the absolute risk approach but have a systolic blood pressure of greater than 160mmHG	5.9mmHg reduction in systolic blood pressure	[2] Law et al. Use of blood pressure lowering drugs in the prevention of cardiovascular disease: meta-analysis of 147 randomised trials in the context of expectations from prospective epidemiological studies. BMJ 2009;338:b1665
8	Blood pressure lowering treatment for those with SBP > 140 mmHG and total CVD risk < 20%	Blood pressure lowering drug therapy for those who do not qualify for preventive measures based on the absolute risk approach but have a systolic blood pressure of greater than 140mmHG	5.9mmHg reduction in systolic blood pressure	[2] Law et al. Use of blood pressure lowering drugs in the prevention of cardiovascular disease: meta-analysis of 147 randomised trials in the context of expectations from prospective epidemiological studies. BMJ 2009;338:b1665
9	Cholesterol lowering treatment for those with Chol > 8 mmol/L and total CVD risk < 20%	Cholesterol lowering drug therapy for those who do not qualify for preventive measures based on the absolute risk approach but have total blood cholesterol of greater than 8 mmol/L	1.05 mmol/L change in cholesterol	[1] Taylor F, et al Statins for the primary prevention of cardiovascular disease. Cochrane Database of Systematic Reviews 2013, Issue 1.
10	Cholesterol lowering treatment for those with Chol > 6 mmol/L and total CVD risk < 20%	Cholesterol lowering drug therapy for those who do not qualify for preventive measures based on the absolute risk approach but have total blood cholesterol of greater than 6 mmol/L	1.05 mmol/L change in cholesterol	[1] Taylor F, et al Statins for the primary prevention of cardiovascular disease. Cochrane Database of Systematic Reviews 2013, Issue 1.
11	Treatment of new cases of acute myocardial infarction with acetylsalicylic acid	Treatment of new cases of acute myocardial infarction with acetylsalicylic acid for the prevention of secondary events	reduction in CVD mortality 15%, ischemic stroke mortality 30%, haemorrhagic stroke mortality 20%	[3] Antithrombotic Trialists' Collaboration. Collaborative meta-analysis of randomised trials of antiplatelet therapy for prevention of death, myocardial infarction, and stroke in high risk patients BMJ. 2002 Jan 12;324(7329):71-86.
12	Combination treatment of new cases of acute myocardial infarction	Drug therapy and counselling to individuals who have had a heart attack	1.05 mmol/L change in cholesterol<br>5.9mmHg reduction in systolic blood pressure	[1] Taylor F, et al Statins for the primary prevention of cardiovascular disease. Cochrane Database of Systematic Reviews 2013, Issue 1.<br>[2] Law et al. Use of blood pressure lowering drugs in the prevention of cardiovascular disease: meta-analysis of 147 randomised trials in the context of expectations from prospective epidemiological studies. BMJ 2009;338:b1665
13	Combination treatment of new cases of stroke	Drug therapy and counselling to individuals who have had a stroke	1.05 mmol/L change in cholesterol<br>5.9mmHg reduction in systolic blood pressure	[1] Taylor F, et al Statins for the primary prevention of cardiovascular disease. Cochrane Database of Systematic Reviews 2013, Issue 1.<br>[2] Law et al. Use of blood pressure lowering drugs in the prevention of cardiovascular disease: meta-analysis of 147 randomised trials in the context of expectations from prospective epidemiological studies. BMJ 2009;338:b1665
	**Diabetes**			

14	Standard Glycaemic control	Effective glycaemic control for people with diabetes, along with standard home glucose monitoring for people on insulin treatment to reduce diabetes complications	Reduces retinopathy incidence by 95% compared to no treatment, and neuropathy by 70-85% depending on intensity of treatment using the approach promoted by Eastman et al[1].	[4] Eastman R.C., et al., 57. Model of complications of NIDDM. I. Model construction and assumptions. Diabetes Care, Volume 20, number 5: 725-43, 199
15	Intensive glycaemic control	Effective glycaemic control for people with uncontrolled diabetes, along with standard home glucose monitoring for people on insulin treatment to reduce diabetes complications	Reduces retinopathy incidence by 95% compared to no treatment, and neuropathy by 70-85% depending on intensity of treatment using the approach promoted by Eastman et al[1].	[4] Eastman R.C., et al., 57. Model of complications of NIDDM. I. Model construction and assumptions. Diabetes Care, Volume 20, number 5: 725-43, 199
16	Retinopathy Screening + photocoagulation	Diabetic retinopathy screening for all diabetes patients every 2 years and laser photocoagulation for prevention of blindness	Reduces by 80% blindness due to retinopathy [1]	[5] American Diabetes Association. Diabetic Retinopathy. Diabetes Care 25 (Suppl 1) 2002
17	Neuropathy screening and preventive foot care	Preventive foot care for people with diabetes (including educational programmes, access to appropriate footwear, multidisciplinary clinics)	Reduces by 50% lower extremity amputation [1]	[6] Apelqvist, J. and J. Larsson, What is the most effective way to reduce incidence of amputation in the diabetic foot? Diabetes Metab Res Rev, 2000. 16 Suppl 1: p. S75-83
	**Asthma**			
18	Inhaled short acting beta agonist (SABA) for intermittent asthma	Step 1: Inhaled short acting beta agonist for intermittent asthma	Change in Disability Weight of 0.01, calculated using Cohen's formula and the Hedges correction factor	Busse et al. Efficacy, tolerability, and effect on asthma-related quality of life of formoterol bid via multidose dry powder inhaler and albuterol QID via metered dose inhaler in patients with persistent asthma: a multicentre, randomized, double-blind, double-dummy, placebo-controlled, parallel-group study. Clinical Therapeutics 2004 http://dx.doi.org/10.1016/j.clinthera.2004.10.004
19	Low dose inhaled beclomethasone plus SABA	Step 2: Inhaled salbutamol prn plus low-dose inhaled beclomethasone	Change in Disability Weight of 0.08, calculated using Cohen's formula and the Hedges correction factor	Adams NP et al. Inhaled beclomethasone versus placebo for chronic asthma. Cochrane Database of Systematic Reviews, 25 January 2005, (1):CD002738.<br>Malmstrom et al. Oral Montelukast, Inhaled Beclomethasone, and Placebo for Chronic Asthma. A Randomized, Controlled Trial. Annals of Internal Medicine, 1999. 130 (6) 487-95
20	High dose inhaled beclomethasone +SABA	Step 3: Same as step 2, but give higher doses of inhaled beclomethasone	Change in Disability Weight of 0.133, calculated using Cohen's formula and the Hedges correction factor	Adams NP et al. Inhaled beclomethasone versus placebo for chronic asthma. Cochrane Database of Systematic Reviews, 25 January 2005, (1):CD002738.
21	Theophylline + High dose inhaled beclomethasone +SABA	Step 4: Add low-dose oral theophylline to Step 3 treatment	Change in Disability Weight of 0.14, calculated using Cohen's formula and the Hedges correction factor	Ukena D, Harnest U, Sakalauskas R, *et al.* Comparison of addition of theophylline to inhaled steroid with doubling of the dose of inhaled steroid in asthma. Eur Respir J 1997;10:2754–2760.
22	Oral Prednisolone + Theophylline + High dose inhaled beclomethasone +SABA	Step 5: Add oral prednisolone	Change in Disability Weight of 0.24, calculated using Cohen's formula and the Hedges correction factor	Chang et al. A 5- versus 3-day course of oral corticosteroids for children with asthma exacerbations who are not hospitalised: a randomised controlled trial. Med J Aust 2008; 189 (6): 306-310.
	**COPD**			

23	Smoking cessation brief intervention by a GP	Brief intervention from physician	Change in Disability weight of 4% plus mortality impact of 15%	Busit AS. The Lung Health Study, Respir ology. 1997. 2(4):303-7
24	Inhaled salbutamol	2 puffs as required, up to four times daily	Change in disability weight of 15%	Sestini P et al. Short-acting beta 2 agonists for stable chronic obstructive pulmonary disease. Cochrane Database of Systematic Reviews, 2002, (4):CD001497.
25	Low-dose oral theophylline	Add low dose (400 mg) theophylline	Change in disability weight of 11%	Ram FS et al. Oral theophylline for chronic obstructive pulmonary disease. Cochrane 2002.
26	Ipratropium inhaler	Ipratropium Bromide 20 mcg inhaler 4 x daily	Change in disability weight o17%	Sestini P et al. Short-acting beta 2 agonists for stable chronic obstructive pulmonary disease. Cochrane Database of Systematic Reviews, 2002, (4):CD001497.
27	Antibiotics	Amoxicillin 500mg 2 x day for 7 days	Change in mortality of 76%	Puhan et al. Exacerbations of chronic obstructive pulmonary disease: when are antibiotics indicated? A systematic review. Respir Res. 2007 Apr 4;8:30.
28	Oral prednisolone	40mg day for seven days	Change in disability weight of 34%	Rodríguez-Roisin, R.COPD exacerbations · 5: Management. Thorax. 2006 Jun; 61(6): 535–544. doi: 10.1136/thx.2005.041863
29	Oxygen, concentration 24-28%	by a mask that limits the concentration to 24-28%	Change in disability weight of 42% plus mortality impact of 50%	Croxton, T. L.;Bailey, W. C. Long-term oxygen treatment in chronic obstructive pulmonary disease: recommendations for future research: an NHLBI workshop report. Am J Respir Crit Care Med. 2006 Aug 15;174(4):373-8. Epub 2006 Apr 13.<br>Nonoyama ML et al. Effect of oxygen on health quality of life in patients with chronic obstructive pulmonary disease with transient exertional hypoxemia. Am J Respir Crit Care Med. 2007 Aug 15;176(4):343-9. Epub 2007 Apr 19.
	**Cervical Cancer**			
30	Basic palliative care for Cervical cancer	home-based and hospital care with multi-disciplinary team and access to opiates and essential supportive medicines	Quality of life increases associated with symptom management	World Health Organization. Planning and implementing palliative care services: a guide for programme managers. 2016. http://apps.who.int/iris/bitstream/10665/250584/1/9789241565417-eng.pdf?ua=1
31	Diagnosis & treatment of cervical cancer stages I and II	Treatment of cervical cancer stages I and II with either surgery or radiotherapy +/- chemotherapy, incl. diagnosis, staging, treatment and post surveillance after completion of treatment	Reduction in mortality associated with treatment, dependent on stage at diagnosis:<br>Stage 1: 77.5%<br>Stage 2: 68.4%<br>Stage 3: 65.0%<br>Stage 4: 75.0%	Goldie S, Grima D, Kohli M, Wright T, Weinstein M, Franco E. A comprehensive natural history model of HPV infection and cervical cancer to estimate the clinical impact of a prophylactic HPV-16/18 vaccine. International Journal of Cancer. 2003;106(6):896-904.<br>National Comprehensive Cancer Network. Cervical Cancer: Clinical Practice Guidelines in Oncology (NCCN Guidelines), Version 1. 2017. https://www.nccn.org/professionals/physician_gls/f_guidelines.asp<br>Chuang L, Temin S, Camacho R, Dueñas-Gonzalez A, Feldman S, Gultekin M et al. Management and Care of Women With Invasive Cervical Cancer: American Society of Clinical Oncology Resource-Stratified<br>Clinical Practice Guideline. Journal of Global Oncology. 2016;2(5):311-340

32	HPV vaccination (2 doses) for preventing cervical cancer	2 doses in girls aged 9-13	Reduction in incidence of 90%	Efficacy of HPV vaccinator in adolescent girls [Internet]. World Health Organisation. 2014 [cited 7 April 2017]. Available from: http://www.who.int/immunization/position_papers/hpv_grad_efficacy_young_females.pdf?ua=1<br>World Health Organisation. Human papillomavirus vaccines: WHO position paper, October 2014. Geneva: WHO Press; 2014 p. No. 43, 2014, 89, 465–492
33	Prevention of cervical cancer through screening with HPV test	Prevention of cervical cancer by screening women aged 30–49 through Human papillomavirus DNA test every 5 years linked with timely treatment of pre-cancerous lesions	Sensitivity: 0.88<br>Specificity: 0.75	World Health Organization. Comprehensive Cervical Cancer Control - A guide to Essential Practice. Geneva: WHO Press; 2014<br>International Agency for Research on Cancer, World Health Organisation. Cervix Cancer Screening. Lyon: IARC Press; 2005.<br>Goldie S, Kuhn L, Denny L, Pollack A, Wright T. Policy Analysis of Cervical Cancer Screening Strategies in Low-Resource Settings. JAMA. 2001;285(24):3107.
34	Prevention of cervical cancer through screening with PAP	Prevention of cervical cancer by screening women aged 30–49 through "Pap" smear (cervical cytology) every 3 years linked with timely treatment of pre-cancerous lesions	Sensitivity 0.62<br>Specificity 0.95	International Agency for Research on Cancer, World Health Organisation. Cervix Cancer Screening. Lyon: IARC Press; 2005.<br>Goldie S, Kuhn L, Denny L, Pollack A, Wright T. Policy Analysis of Cervical Cancer Screening Strategies in Low-Resource Settings. JAMA. 2001;285(24):3107.
35	Prevention of cervical cancer through screening with VIA	Prevention of cervical cancer by screening women aged 30–49 through visual inspection with acetic acid every 3 years linked with timely treatment of pre-cancerous lesions	Sensitivity 0.66<br>Specificity: 0.77	International Agency for Research on Cancer, World Health Organisation. Cervix Cancer Screening. Lyon: IARC Press; 2005.<br>Goldie S, Kuhn L, Denny L, Pollack A, Wright T. Policy Analysis of Cervical Cancer Screening Strategies in Low-Resource Settings. JAMA. 2001;285(24):3107.
	**Colorectal Cancer**			
36	Basic palliative care for Colorectal Cancer	home-based and hospital care with multi-disciplinary team and access to opiates and essential supportive medicines	Quality of life increases associated with symptom management	World Health Organization. Planning and implementing palliative care services: a guide for programme managers. 2016. http://apps.who.int/iris/bitstream/10665/250584/1/9789241565417-eng.pdf?ua=1

37	Diagnosis & treatment of colorectal cancer stages I and II	Treatment of colorectal cancer stages I and II with surgery +/- chemotherapy and radiotherapy, incl. diagnosis, staging, treatment and surveillance after completion of treatment	Reduction in mortality associated with treatment, dependent on stage at diagnosis:<br>Stage 1: 94.4% decrease<br>Stage 2: 94.4% decrease<br>Stage 3: 91.4% decrease<br>Stage 4: 36.7.0% decrease	Liu CY, Chen WTL, Kun PT, Chiu CF, Wang YH, Shieh SH,Tsai WC. Characteristics, survival, and related factors of newly diagnosed colorectal cancer patients refusing cancer treatments under a universal health insurance program. BMC Cancer. 2014; 14(446).<br>National Comprehensive Cancer Network. Colon Cancer: Clinical Practice Guidelines in Oncology (NCCN Guidelines), Version 2. 2017. https://www.nccn.org/professionals/physician_gls/f_guidelines.asp<br>Frazier AL, Colditz GA, Fuchs CS, and et al. Cost-effectiveness of Screening for Colorectal Cancer in the General Population. JAMA. 2000; 284(15): 1954-1961.<br>Wu GHM, Wang YM, Yen AMF, Wong JM, Lai HC, Warwick J, Chen THHC. Cost-effectiveness analysis of colorectal cancer screening with stool DNA testing in intermediate-incidence countries. BMC Cancer. 2006; 6(136).<br>Chadder J, Dewar R, Shack L , Nishri D, Niu J, Lockwood G. A first look at relative survival by stage for colorectal and lung cancers in Canada. Current Oncology. 24 Apr 2016;23(2): 119-24.<br>Colorectal Cancer Survival by Stage - NCIN Data Briefing. June 2009. http://www.ncin.org.uk/publications/data_briefings/colorectal_cancer_survival_by_stage.
	**Breast Cancer**			
38	Basic palliative care for Breast Cancer:	home-based and hospital care with multi-disciplinary team and access to opiates and essential supportive medicines	Quality of life increases associated with symptom management	World Health Organization. Planning and implementing palliative care services: a guide for<br>programme managers. 2016.<br>http://apps.who.int/iris/bitstream/10665/250584/1/9789241565417-eng.pdf?ua=1

39	Diagnosis & treatment of breast cancer stages I and II	with surgery, radiotherapy and chemotherapy and hormone therapy as needed)	Reduction in mortality associated with treatment, dependent on stage at diagnosis:<br>Stage 1: 95.7% decrease<br>Stage 2: 78.3% decrease<br>Stage 3: 59.6% decrease<br>Stage 4: 46.0% decrease	Zelle SG, Nyarko KM, Bosu WK, Aikins M, Niens LM, Lauer JA, Sepulveda CR, Hontelez JAC, Baltussen R. Costs, effects and cost-effectiveness of breast cancer control in Ghana. Tropical Medicine and International Health. 2012; 17(8): 1031-1043.<br>Groot MT, Baltussen R, Uyl-de Groot CA, Anderson BO, Hortobágyi GN. Costs and Health Effects of Breast Cancer Interventions in Epidemiologically Different Regions of Africa, North America, and Asia. The Breast Journal. 2006; 12 (Supplement s1): S1-S122.<br>Perez E , Romond E, Suman V, Jeong J, Sledge G, Geyer CJ, Martino S, Rastogi P, Gralow J, Swain S, Winer E, Colon-Otero G, Davidson N, Mamounas E, Zujewsk J, Wolmark N. Trastuzumab plus adjuvant chemotherapy for human epidermal growth factor receptor 2-positive breast cancer: planned joint analysis of overall survival from NSABP B-31 and NCCTG N9831. J Clin Oncol. 20 Nov 2014; 32(33): 3744-52.<br>Davies C et al. Long-term effects of continuing adjuvant tamoxifen to 10 years versus stopping at 5 years after diagnosis of oestrogen receptor-positive breast cancer: ATLAS, a randomised trial. The Lancet. 9 March 2013; 381(9869): 805-16.<br>Feng W, Ke Y, Ze-Dong D, Xiao-Feng H, Peng-Fei Z, Rui-Lei T, Qiu L. Cost-effectiveness analysis of colon cancer treatments from MOSIAC and No. 16968 trials. World J Gastroenterol. 21 Dec 2014;20(47):17976-84.
40	Screening with mammography	(once in 2 years for the age group 50 to 69 years) linked with timely diagnosis and treatment	Sensitivity rate of 0.76, specificity rate of 0.93 for twice yearly screening	International Agency for Research on Cancer. Breast Cancer Screening: IARC Handbook of Cancer Prevention. 2016;15
	**Tobacco**			
41	Protect people from tobacco smoke	Eliminate exposure to second-hand tobacco smoke in all indoor workplaces, public places, public transport	Reduction in prevalence of 4% if implemented at the highest intensity level	Levy et al. The Impact of Implementing Tobacco Control Policies: An Update and Extension of the Tobacco Control Scorecard. [28]
42	Offer help to quit tobacco use	Provide cost-covered, effective and population-wide support (including brief advice and national toll-free quit line services) for tobacco cessation to all those who want to quit	Reduction in prevalence of 5.5% - 11% if implemented at the highest intensity level	Levy et al. The Impact of Implementing Tobacco Control Policies: An Update and Extension of the Tobacco Control Scorecard. [28]
43	Warn about the dangers of tobacco, 1	Implement large graphic health warnings on all tobacco packages	Reduction in prevalence of 4% if graphic health warnings implemented at the highest intensity level	Levy et al. The Impact of Implementing Tobacco Control Policies: An Update and Extension of the Tobacco Control Scorecard. [28]

44	Warn about the dangers of tobacco, 2	Additionally, implement plain/standardized packaging	Reduction in prevalence of 0.5 - 3.8% attributable to plain packaging, when implemented as part of a comprehensive approach to tobacco control, including graphic health warnings implemented at the highest intensity level.	The illustrative 0.5% figure is derived from the cost-benefit analysis in Post-Implementation Review Tobacco Plain Packaging, 2016, Australian Government, Department of Health, 26 February 2016, para. 166 available at http://ris.pmc.gov.au/2016/02/26/tobacco-plain-packaging. The 3.8% figure is a median estimate of the drop in adult smoking prevalence relied upon in the United Kingdom Impact Assessment: Standardised Packaging of Tobacco Products: Impact Assessment, para. 219 available at https://www.gov.uk/government/consultations/standardised-packaging-of-tobacco-products-draft-regulations
45	Enforce bans on tobacco advertising, promotion and sponsorship	Enact and enforce comprehensive bans on tobacco advertising, promotion and sponsorship	Reduction in prevalence of 10% if implemented at the highest intensity level	Levy et al. The Impact of Implementing Tobacco Control Policies: An Update and Extension of the Tobacco Control Scorecard. [28]
46	Raise taxes on tobacco	Increase excise taxes and prices on tobacco products	Elasticity is -0.2 to- 0.5. Analysis undertaken based on an assumed tax increase that increases the retail price of cigarettes by 25%.	IARC HANDBOOKS OF CANCER PREVENTION Tobacco Control
47	Mass media campaigns for smoking prevention	Implement effective mass media campaigns that educate the public about the harms of smoking/tobacco use and second hand smoke	Reduction in prevalence of 3.8% if implemented at the highest intensity level	Levy et al. The Impact of Implementing Tobacco Control Policies: An Update and Extension of the Tobacco Control Scorecard. [28]
	**Alcohol**			
48	Increase in excise taxes on alcoholic beverages	50% increase in excise taxes on alcoholic beverages	Beverage-specific demand elasticities for alcohol, by country income level, based on international reviews (range -0.3 [beer, HIC] to -0.79 [wine and spirits, LMIC)	Fogarty J. The demand for beer, wine and spirits: a survey of the literature. Journal of Economic Surveys 2010, 24(3):428-478. Sornpaisarn B et al (2013). Elasticity of alcohol consumption, alcohol-related harms, and drinking initiation in low-and middle-income countries: a systematic review and meta-analysis. International Journal of Alcohol and Drug Research, 2013; 2 (1): 45-58.
49	Restrictions on marketing of alcoholic beverages	Enforcement of bans or comprehensive restrictions on exposure to alcohol advertising, promotion and sponsorship (across multiple types of media)	1.2% reduction in prevalence, based on cross-sectional analyses of data from 15 LAMICs, which found an inverse association between increased marketing restrictions and total drinking volume (a 3% reduction in drinking volume per additional level of restriction for beer, wine and spirits across 4 types of media respectively, for a total effect size of -0.72 for a 2-point increased restriction level)	Cook WK, Bond J, Greenfield TK. Are alcohol policies associated with alcohol consumption in Low- and Middle-income countries? Addiction 2014, 109(7):1081-1090.

50	Restrictions on the physical availability of retailed alcohol	Enforcement of restrictions on the physical availability of retailed alcohol (via reduced hours of sale)	1.8-2.1% (male), 4% (female) reduction in prevalence, based on cross-sectional analyses of data from 15 LAMICs, which found an inverse association between increased restrictions on business hours for off-premises alcohol sales and total drinking volume (-0.88)	Cook WK, Bond J, Greenfield TK. Are alcohol policies associated with alcohol consumption in Low- and Middle-income countries? Addiction 2014, 109(7):1081-1090.
51	Enforcement of drink-driving laws and blood alcohol concentration limits via sobriety checkpoints	Enforcement of drink-driving laws and blood alcohol concentration limits via sobriety checkpoints	15-20% reduction in alcohol-attributable years lived with disability (YLD) and road traffic deaths, respectively	Elvik R (2009). Handbook of Road Safety Measures. Emerald publishing group.
52	Provision of brief psychosocial intervention for persons with hazardous and harmful alcohol use	Provision of brief psychosocial intervention for persons with hazardous and harmful alcohol use; Intervention coverage modelled at 50%.	Prevalence reduction varies by age, sex and region (0% [female, 15-59 years], 11-17% [female, 60+ years], 13-21% [male, 15-59 years], 6-11% [males, 60+ years]), based on change in consumption (3.6 drinks per week less) and heavy episodic drinking (12% less).	Jonas DE et al. Screening, Behavioral Counseling, and Referral in Primary Care To Reduce Alcohol Misuse. Rockville (MD): Agency for Healthcare Research and Quality (US); 2012 Jul.
	**Physical Inactivity**			
53	Provide physical activity counselling as part of routine primary health care services through the use of a brief intervention	a 2 minute brief intervention from a GP or primary care giver.	The number needed to treat with an intervention for one additional sedentary adult to meet internationally recommended levels of activity at 12 months was 12 (7 to 33)	Orrow Gillian, Kinmonth Ann-Louise, SandersonSimon, Sutton Stephen. Effectiveness of physical activity promotion based in primary care: systematic review and meta-analysis of randomised controlled trials *BMJ* 2012; 344 :e1389
54	Implement community wide public education and awareness campaign for physical activity	Implement community wide public education and awareness campaign for physical activity which includes a mass media campaign combined with other community based education, motivational and environmental programs aimed at supporting behavioural change of physical activity levels	5.2% reduction in the population who do not meet physical activity recommendations	Justine E. Leavy, et al Effects of Find 30 Every Day. *Health Education & Behavior* Vol 40, Issue 4, pp. 480 - 492
	**Unhealthy diet**			
55	Harness the Industry for voluntary reformulation (salt)	Reduce salt intake by engaging the industry in a voluntary reformulation process	2.2 g/day salt reduction	Menos Sal + Vida. Buenos Aires: Ministry of Health; 2015 (http://www.msal.gob.ar/ent/index.php/informacion-para-ciudadanos/menos-sal--vida).
56	Adopt standards for front of pack labelling	Reduce salt intake through implementation of front-of-pack labelling	1.8g/day salt reduction men, 1.0 g/day salt reduction women	Laatikainen T, Pietinen P, Valsta L, Sundvall J, Reinivuo H, Tuomilehto J. Sodium in the Finnish diet: 20-year trends in urinary sodium excretion among the adult population. Eur J Clin Nutr 2006; 60: 965–970

57	Knowledge: Education and communication	Reduce salt intake through a behaviour change communication mass media campaign	5% reduction in salt intake per day	Do, Santos, Trieu, et al. Effectiveness of a Communication for Behavioral Impact (COMBI) Intervention to Reduce Salt Intake in a Vietnamese Province Based on Estimations From Spot Urine Samples. J Clin Hypertens. 18 (11):1135-1142
58	Environment: Salt reduction strategies in community based eating spaces	Reduce salt intake through establishment of a supportive environment in public institutions such as hospitals, schools and nursing homes to enable low sodium meals to be provided	7% reduction in salt intake per day	[1] Nelson M, Nicholas, J., Haroun, D., Harper, C., Wood, L., Storey, C., Pearce, J. The impact of school food standards on children's eating habits in England. Improving diets and nutrition: food-based approaches. Rome, Italy: Food and Agriculture Organization of the United Nations; 2014. p. 137<br>Grimes CA, Campbell KJ, Riddell LJ, Nowson CA. Sources of sodium in Australian children's diets and the effect of the application of sodium targets to food products to reduce sodium intake. Br J Nutr. 2011 Feb;105(3):468-77
59	Trans fat elimination	Complete elimination of industrial trans fats through the development of legislation banning their use in the food chain	Reduction in CVD mortality of 13 deaths per 100,000	Restrepo BJ et al. Trans fat and cardiovascular disease mortality: Evidence from bans in restaurants in New York. J Health Econ. 2016 Jan;45:176-96
	**Anxiety**			
60	Basic psychosocial treatment for mild cases of anxiety disorder	Basic psychosocial treatment for mild cases of anxiety disorder	60% improvement in remission, 12.3% improvement in functioning	Chisholm, D., K. Sweeny, P. Sheehan, B. Rasmussen, F. Smit, P., Cuijpers, S. Saxena (2016). Scaling up treatment of depression and anxiety: a global return on investment analysis. Lancet Psychiatry, 3: 415-424.
61	Basic psychosocial and anti-depressant drug treatment for moderate-severe cases of anxiety disorder	Basic psychosocial and anti-depressant drug treatment for moderate-severe cases of anxiety disorder	60% improvement in remission, 15.3% improvement in functioning	
62	Intensive psychosocial and anti-depressant drug treatment for moderate-severe cases of anxiety disorder	Intensive psychosocial and anti-depressant drug treatment for moderate-severe cases of anxiety disorder	60% improvement in remission, 17.2% improvement in functioning	
	**Depression**			
63	Basic psychosocial treatment for mild cases of depression	Basic psychosocial treatment for mild cases of depression	25% improvement in remission, 4.9% improvement in functioning	Chisholm, D., K. Sweeny, P. Sheehan, B. Rasmussen, F. Smit, P., Cuijpers, S. Saxena (2016). Scaling up treatment of depression and anxiety: a global return on investment analysis. Lancet Psychiatry, 3: 415-424.
64	Basic psychosocial treatment and anti-depressant medication for first-episode moderate-severe cases of depression	Basic psychosocial treatment and anti-depressant medication for first-episode moderate-severe cases of depression	35% improvement in remission, 7.7% improvement in functioning	
65	Intensive psychosocial treatment and anti-depressant medication for first-episode moderate-severe cases of depression	Intensive psychosocial treatment and anti-depressant medication for first-episode moderate-severe cases of depression	35% improvement in remission, 10.5% improvement in functioning	
66	Intensive psychosocial treatment and anti-depressant medication for recurrent moderate-severe cases of depression on an episodic basis	Intensive psychosocial treatment and anti-depressant medication for recurrent moderate-severe cases of depression on an episodic basis	35% improvement in remission, 10.5% improvement in functioning	

67	Intensive psychosocial treatment and anti-depressant medication for recurrent moderate-severe cases of depression on a maintenance basis	Intensive psychosocial treatment and anti-depressant medication for recurrent moderate-severe cases of depression on a maintenance basis	35% improvement in remission, 10.5% improvement in functioning	
	**Bipolar disorder**			
68	Mood-Stabilizing Medication + Basic Psychosocial treatment for bipolar disorder (older drugs)	Older mood-stabilising medication (lithium) and basic psychosocial treatment	24% improvement in functioning, plus 65% reduction in case fatality rate	Chisholm D, Van Ommeren M, Ayuso-Mateos JL, Saxena S (2005). Cost-effectiveness of clinical interventions for reducing the burden of bipolar disorder: a global analysis (WHO-CHOICE). British Journal of Psychiatry, 187: 559-67.
69	Mood-Stabilizing Medication + Intensive Psychosocial treatment for bipolar disorder (older drugs)	Older mood-stabilising medication (lithium) and intensive psychosocial treatment	24% improvement in functioning, plus 65% reduction in case fatality rate	
70	Mood-Stabilizing Medication + Basic Psychosocial treatment for bipolar disorder (newer drugs)	Newer mood-stabilising medication (valproic acid) and basic psychosocial treatment	24% improvement in functioning	
71	Mood-Stabilizing Medication + Intensive Psychosocial treatment for bipolar disorder (newer drugs)	Newer mood-stabilising medication (valproic acid) and intensive psychosocial treatment	24% improvement in functioning	
	**Psychosis**			
72	Antipsychotic Medication + Basic Psychosocial treatment of psychosis (older drugs)	Older (neuroleptic) medication and basic psychosocial treatment	21.8% improvement to functioning, based on effect size of 0.5	Chisholm D, Gureje O, Saldivia S, Villalón Calderón M, Wickremasinghe R, Mendis N, Ayuso-Mateos JL, Saxena S (2008). Schizophrenia treatment in the developing world: an inter-regional and multi-national cost-effectiveness analysis. Bulletin of the World Health Organization, 86: 542-551.
73	Antipsychotic Medication + Intensive Psychosocial treatment of psychosis (older drugs)	Older (neuroleptic) medication and intensive psychosocial treatment (e.g. family therapy)	22.6% improvement to functioning, based on effect size of 0.495	
74	Antipsychotic Medication + Basic Psychosocial treatment of psychosis (newer drugs)	Newer anti-psychotic medication and basic psychosocial treatment	39% improvement to functioning, based on effect size of 0.855	
75	Antipsychotic Medication + Intensive Psychosocial treatment of psychosis (newer drugs)	Newer anti-psychotic medication and intensive psychosocial treatment (e.g. family therapy)	40.3% improvement to functioning, based on effect size of 0.885	
	**Epilepsy**			
76	Antiepileptic Medication + Basic Psychosocial treatment of epilepsy (older drugs)	Older anti-epileptic medication (e.g. phenobarbital, phenytoin) + basic psychosocial treatment	60% improvement in remission, 47% improvement in functioning	Chisholm D (2005). Cost-effectiveness of first-line anti-epileptic drug treatments in the developing world: a population-level analysis. Epilepsia, 46: 751-9.
77	Antiepileptic Medication + Basic Psychosocial treatment of epilepsy (newer drugs)	Newer anti-epileptic medication (e.g. valproate, carbamazepine) + basic psychosocial treatment	60% improvement in remission, 47% improvement in functioning	
