# Supplementary material for: Cost-Effectiveness of Population Level and Individual Level Interventions to Combat Non-communicable Disease in Eastern Sub-Saharan Africa and South East Asia: A WHO-CHOICE Analysis
Source: Int J Health Policy Manag. 2021 Jun 7;10(11):724–33. doi: 10.34172/ijhpm.2021.37 (PMC9278376; doi:10.34172/ijhpm.2021.37)
Supplement: Supplementary file 2 — Costing Inputs for NCD Interventions. [file ijhpm-10-724-s002.pdf]

Article title: Cost-Effectiveness of Population Level and Individual Level Interventions to Combat Non-communicable Disease in Eastern Sub-Saharan Africa and South East Asia: A WHO-CHOICE Analysis

Journal name: International Journal of Health Policy and Management (IJHPM)

Authors' information: Melanie Y. Bertram[1]*, Daniel Chisholm[2], Rory Watts[1], Temo Waqanivalu[3], Vinayak Prasad[3], Cherian Varghese[4]

[1]Department of Health Systems Governance and Financing, World Health Organization, Geneva, Switzerland.

[2]Department of Mental Health and Substance Abuse, World Health Organization, Geneva, Switzerland.

[3]Department of Prevention of Non-Communicable Diseases, World Health Organization, Geneva, Switzerland.

[4]Department of Management of Non Communicable Diseases, Violence and Injury, World Health Organization, Geneva, Switzerland.

(*Corresponding author: bertramm@who.int)

Supplementary file 2. Costing Inputs for NCD Interventions

Number	Disease /Intervention	Costing inputs			
	**Cardiovascular Disease**	**Patient level costs**	**Inpatient Bed days**	**Outpatient visits**	**Staff**
1	Combination therapy for prevention of CVD in those with 30%+ risk[9]	Combination of: Hydrochlorothiazide, tablet, 25 mg;<br>Enalapril, tablet, 20 mg;<br>Atenolol, tablets, 50 mg;<br>Amlodipine, tablet, 10 mg;<br>Simvastatin, 15 mg;<br>Blood glucose level test;<br>Cholesterol test;<br>Urine sugar analysis		4 visits	10 minutes with doctor. 20 minutes with nurse for 7% of population with comorbid diabetes
2	Blood pressure lowering treatment for those with SBP > 160 mmHG and total CVD risk < 30%[9]	Hydrochlorothiazide, tablet, 25 mg;<br>Enalapril, tablet, 20 mg;<br>Atenolol, tablets, 50 mg;<br>Amlodipine, tablet, 10 mg;<br>Blood glucose level test;<br>Cholesterol test;<br>Urine sugar analysis		3 visits	10 minutes with doctor. 20 minutes with nurse.

3	Blood pressure lowering treatment for those with SBP > 140 mmHG and total CVD risk < 30%[9]	Hydrochlorothiazide, tablet, 25 mg;<br>Enalapril, tablet, 20 mg;<br>Atenolol, tablets, 50 mg;<br>Amlodipine, tablet, 10 mg;<br>Blood glucose level test;<br>Cholesterol test;<br>Urine sugar analysis		3 visits	10 minutes with doctor. 20 minutes with nurse.
4	Cholesterol lowering treatment for those with Chol > 8 mmol/L and total CVD risk < 30%[9]	Simvastatin, 15 mg;<br>Blood glucose level test;<br>Cholesterol test;<br>Urine sugar analysis		3 visits	10 minutes with doctor. 20 minutes with nurse.
5	Cholesterol lowering treatment for those with Chol > 6 mmol/L and total CVD risk < 30%[9]	Simvastatin, 15 mg;<br>Blood glucose level test;<br>Cholesterol test;<br>Urine sugar analysis		3 visits	10 minutes with doctor. 20 minutes with nurse.
6	Combination therapy for prevention of CVD in those with 20%+ risk[9]	Combination of: Hydrochlorothiazide, tablet, 25 mg;<br>Enalapril, tablet, 20 mg;<br>Atenolol, tablets, 50 mg;<br>Amlodipine, tablet, 10 mg;<br>Simvastatin, 15 mg;<br>Blood glucose level test;<br>Cholesterol test;<br>Urine sugar analysis		4 visits	10 minutes with doctor. 20 minutes with nurse for 7% of population with comorbid diabetes
7	Blood pressure lowering treatment for those with SBP > 160 mmHG and total CVD risk < 20%[9]	Hydrochlorothiazide, tablet, 25 mg;<br>Enalapril, tablet, 20 mg;<br>Atenolol, tablets, 50 mg;<br>Amlodipine, tablet, 10 mg;<br>Blood glucose level test;<br>Cholesterol test;<br>Urine sugar analysis		3 visits	10 minutes with doctor. 20 minutes with nurse.
8	Blood pressure lowering treatment for those with SBP > 140 mmHG and total CVD risk < 20%[9]	Hydrochlorothiazide, tablet, 25 mg;<br>Enalapril, tablet, 20 mg;<br>Atenolol, tablets, 50 mg;<br>Amlodipine, tablet, 10 mg;<br>Blood glucose level test;<br>Cholesterol test;<br>Urine sugar analysis		3 visits	10 minutes with doctor. 20 minutes with nurse.

9	Cholesterol lowering treatment for those with Chol > 8 mmol/L and total CVD risk < 20%[9]	Simvastatin, 15 mg;<br>Blood glucose level test;<br>Cholesterol test;<br>Urine sugar analysis		3 visits	10 minutes with doctor. 20 minutes with nurse.
10	Cholesterol lowering treatment for those with Chol > 6 mmol/L and total CVD risk < 20%[9]	Simvastatin, 15 mg;<br>Blood glucose level test;<br>Cholesterol test;<br>Urine sugar analysis		3 visits	10 minutes with doctor. 20 minutes with nurse.
11	Treatment of new cases of acute myocardial infarction with acetylsalicylic acid[9]	Acetyl salicylic acid (aspirin), tablet, 75 mg;<br>Oxygen, 1000 litres, primarily with oxygen cylinders;<br>IV line;<br>Saline solution	2 days immediately post event	5 visits	20 minutes with doctor. 4 visits of 10 minutes with nurse.
12	Combination treatment of new cases of acute myocardial infarction [9]	Acetyl salicylic acid (aspirin), tablet, 75 mg;<br>Enalapril, tablet, 20 mg;<br>Atenolol, tablets, 50 mg;<br>Simvastatin, 15 mg;<br>Prednisolone, tablet, 5 mg;<br>Blood glucose level test;<br>Cholesterol test;<br>Urine analysis		6 visits	6 times 10 minutes with doctor. 4 visits with nurse.
13	Combination treatment of new cases of stroke[9]	Acetyl salicylic acid (aspirin), tablet, 75 mg;<br>Enalapril, tablet, 20 mg;<br>Atenolol, tablets, 50 mg;<br>Simvastatin, 15 mg;<br>Prednisolone, tablet, 5 mg;<br>Blood glucose level test;<br>Cholesterol test;<br>Urine analysis	2 days immediately post event	5 visits<br>4 additional outpatient visits with physiotherapist or nurse for 20% of patients	6 times 10 minutes with doctor. 4 times 20 minutes with physiotherapy nurse for 20% of population.
	**Diabetes**				
14	Standard glycaemic control[9]	Insulin in 1/3 of patients<br>Home glucose monitoring;<br>Glibenclamide, 5 mg tab;<br>Metformin, 850 mg tab;<br>HbA1c test;<br>Blood test: Test for fasting lipid profile ;<br>Urine test: Test for urine albumin excretion and albumin to creatinine ratio		4 visits	4 times 10 minute nurse visits, 1 GP visit per year, for 10% of patients 1 specialist visit per year

15	Intensive glycaemic control[9]	Insulin for 1/2 of patients;<br>Home glucose monitoring;<br>Glibenclamide, 5 mg tab;<br>Metformin, 850 mg tab;<br>HbA1c test;<br>Blood test: Test for fasting lipid profile;<br>Urine test: Test for urine albumin excretion and albumin to creatinine ratio		4 visits	4 times 10 minute nurse visits, 2 GP visit per year, 1 specialist visit per year
16	Retinopathy Screening + photocoagulation[9]	Fluorescein drops;<br>Portable slit lamp;<br>Laser Photocoagulation;<br>Tetracaine drops;<br>Antireflective lens		1 visit for screening, 2 visits for follow up care in 5% of patients	1 specialist visit for screening, 2 longer specialist visits for the 5% of patients requiring treatment
17	Neuropathy screening and preventive foot care[9]	Gloves, exam, latex, disposable, pair;<br>Monofilament, 10 g;<br>Protective shoes;<br>Insoles		1 visit for screening, 3 visits for follow up care in 10% of patients	1 specialist visit for screening, 3 longer specialist visits for the 10% of patients requiring treatment
	**Asthma**				
18	Inhaled short acting beta agonist (SABA) for intermittent asthma[9]	Salbutamol inhaler, 100 mcg		3 visits	1 GP visit, 2 nurse visits
19	Low dose inhaled beclomethasone plus SABA[9]	Salbutamol inhaler, 100 mcg;<br>Beclomethasone 100mg		3 visits	1 GP visit, 2 nurse visits
20	High dose inhaled beclomethasone +SABA[9]	Salbutamol inhaler, 100 mcg;<br>Beclomethasone 100mg		3 visits	1 GP visit, 2 nurse visits
21	Theophylline + High dose inhaled beclomethasone +SABA[9]	Salbutamol inhaler, 100 mcg;<br>Beclomethasone 100mg;<br>Theophylline 200mg SR		3 visits	1 GP visit, 2 nurse visits
22	Oral Prednisolone + Theophylline + High dose inhaled beclomethasone +SABA[9]	Salbutamol inhaler, 100 mcg;<br>Beclomethasone 100mg;<br>Theophylline 200mg SR;<br>Prednisolone, tablet, 20 mg		3 visits	1 GP visit, 2 nurse visits
	**COPD**				
23	Smoking cessation brief intervention by a GP[9]			1 visit	1 GP visit

24	Inhaled salbutamol[9]	Salbutamol inhaler, 100 mcg		3 visits	1 GP visit, 2 nurse visits
25	Low-dose oral theophylline[9]	Theophylline 200mg SR		3 visits	1 GP visit, 2 nurse visits
26	Ipratropium inhaler[9]	Ipratropium Bromide 20 mcg inhaler		3 visits	1 GP visit, 2 nurse visits
27	Antibiotics[9]	Amoxicillin, 500 mg tab	7 days		7 GP visits, 14 nurse visits
28	Oral prednisolone[9]	Prednisolone, tablet, 20 mg	8 days		7 GP visits, 14 nurse visits
29	Oxygen, concentration 24-28%[9]	Oxygen, 1000 litres, primarily with oxygen cylinders	9 days		7 GP visits, 14 nurse visits
	**Cervical Cancer**				
30	Basic palliative care for Cervical cancer[29]	Ibuprofen, 400 mg tab;<br>Paracetamol, tablet, 500 mg;<br>Morphine injection (5mL);<br>Morphine oral liquid (10mg/mL);<br>Dexamethasone (4mg tablets);<br>Docusate (100mg tablet);<br>Senna, 7.5 mg tab;<br>Amitriptyline 25 mg tablets;<br>Loperamide 2 mg;<br>Metoclopramide (10mg tablet);<br>Morphine slow release tablet (10mg)	15 days	2 visits	1 visit from Pharmaceutical technician<br>15 visits with a nurse<br>1 long 2 hr visit from a specialist<br>15 visits with a GP

31	Diagnosis & treatment of cervical cancer stages I and II [29]	Formalin, 1 litre;<br>H and E staining;<br>Ultrasound gel;<br>Ultrasound probe cover;<br>X-ray film;<br>X-ray film chemistry;<br>Vaginal estrogen;<br>Dexamethasone, 4 mg, 1 ampoule;<br>Ondansetron (4mg tablets);<br>Cotton swab;<br>Gauze pad, 10 x 10 cm, sterile;<br>Gauze pad, 10X4 cm;<br>Specimen container;<br>Cidex 2-4% glutaraldehyde (cl), 100ml;<br>Lidocaine HCl (in dextrose 7.5%), ampoule 2 ml;<br>Needle, spinal, 22g (disposable);<br>Syringe, 10 cc with needle;<br>Syringe, 5 cc with needle;<br>Syringes, single use, 20 ml;<br>Skin preparatory solution, 500 ml vial;<br>Sponges;<br>Sutures;<br>Scalpel blade, disposable;<br>Electrosurgical pen;<br>Gloves, exam, latex, disposable, pair;<br>Syringe, needle + swab;<br>Perthodine (cl);<br>Cannula, IV, 22G, sterile, disposable ;<br>Definitive radiotherapy (50Gy in 25 fractions);<br>Cisplatin (50 mg tab);<br>IV giving/infusion set, with needle;<br>Cannulae;<br>Complete blood count;<br>Blood collecting tube, 5 ml;<br>Total bilirubin;<br>Alkaline phosphatase reagents;<br>Electrolytes test;<br>BUN test;<br>Serum creatinine	2days	5 visits	Nurses - 5 visits<br>Ob\Gyns - 5 visits<br>Laboratory technicians/assistants - 2 hrs<br>Assistant nurses and midwives 2 visits<br>Pharmaceutical technicians/assistants 2 visits<br>Other specialist doctors 2 visits<br>Radiographers/X-ray technicians 2 extra-long consults
32	HPV vaccination (2 doses) for preventing cervical cancer[29]	Syringe, auto-disposable, 0.5 ml, with needle<br>Safety box for used syringes/needles, 5 litre<br>HPV vaccine		2 visits	Nurse

33	Prevention of cervical cancer through screening with HPV DNA test[29]	Pipettes, fixed, 50 uL;<br>Pipette, repeater;<br>Pipette tips;<br>Repeater tip;<br>Gloves, exam, latex, disposable, pair;<br>Cotton swab;<br>Specimen container;<br>CareHPV Test Kit;<br>IEC materials;<br>Data collection forms;<br>Azithromycin, 500 mg;<br>Cefrriaxone, powder for infection, 250 ml vial;<br>Syringe, needle + swab		1 visit	Community health worker and nurse
34	Prevention of cervical cancer through screening with PAP[29]	Gloves, exam, latex, disposable, pair;<br>Applicator sticks;<br>Microscope slides with frosted end, pack of 50;<br>Cervical cytology brush/scraper;<br>Stable chlorine disinfectant, pack of 100 tablets;<br>Fixing chemicals;<br>IEC materials;<br>Data collection forms;<br>Azithromycin, 500 mg;<br>Ceftriaxone, powder for infection, 250 ml vial;<br>Syringe, needle + swab		1 visit	Community health worker, assistant nurse, nurse and physician
35	Prevention of cervical cancer through screening with VIA[29]	Gloves, exam, latex, disposable, pair;<br>Acetic acid, 5% dilute, 5 ml;<br>Cotton wool;<br>Applicator sticks;<br>KY jelly packet;<br>Condom, male;<br>Gauze pad, 10 x 10 cm, sterile;<br>Stable chlorine disinfectant, pack of 100 tablets;<br>70 isopropyl alcohol, 10 ml;<br>Soap or hand sanitizer, 1L;<br>IEC materials;<br>Data collection forms;<br>Azithromycin, 500 mg;<br>Ceftriaxone, powder for infection, 250 ml vial;<br>Syringe, needle + swab		1 visit	Community health worker and nurse
	**Colorectal Cancer**				

36	Basic palliative care for Colorectal Cancer[29]	Ibuprofen, 400 mg tab;<br>Paracetamol, tablet, 500 mg;<br>Morphine injection (5mL);<br>Morphine oral liquid (10mg/mL);<br>Dexamethasone (4mg tablets);<br>Docusate (100mg tablet);<br>Senna, 7.5 mg tab;<br>Amitriptyline 25 mg tablets;<br>Loperamide 2 mg;<br>Metoclopramide (10mg tablet);<br>Morphine slow release tablet (10mg)	15 days	2 visits	1 visit from Pharmaceutical technician<br>15 visits with a nurse<br>1 long 2 hr visit from a specialist<br>15 visits with a GP
37	Diagnosis & treatment of colorectal cancer stages I and II [29]	Formalin, 1 litre;<br>H and E staining;<br>Ultrasound gel;<br>Ultrasound probe cover;<br>X-ray film;<br>X-ray film chemistry;<br>Vaginal estrogen;<br>Dexamethasone, 4 mg, 1 ampoule;<br>Ondansetron (4mg tablets);<br>Cotton swab;<br>Gauze pad, 10 x 10 cm, sterile;<br>Gauze pad, 10X4 cm;<br>Specimen container;<br>Cidex 2-4% glutaraldehyde (cl), 100ml;<br>Lidocaine HCl (in dextrose 7.5%), ampoule 2 ml;<br>Needle, spinal, 22g (disposable);<br>Syringe, 10 cc with needle;<br>Syringe, 5 cc with needle;<br>Syringes, single use, 20 ml;<br>Skin preparatory solution, 500 ml vial;<br>Sponges;<br>Sutures;<br>Scalpel blade, disposable;<br>Electrosurgical pen;<br>Gloves, exam, latex, disposable, pair;<br>Syringe, needle + swab;<br>Perthodine (cl);<br>Cannula, IV, 22G, sterile, disposable ;<br>Definitive radiotherapy (50Gy in 25 fractions);<br>Cisplatin (50 mg tab);<br>IV giving/infusion set, with needle;<br>Cannulae;<br>Complete blood count;	2days	5 visits	Nurses - 5 visits<br>Specialist doctor - 5 visits<br>Laboratory technicians/assistants - 2 hrs<br>Assistant nurses and midwives 2 visits<br>Pharmaceutical technicians/assistants 2 visits<br>Other specialist doctors 2 visits<br>Radiographers/X-ray technicians 2 extra-long consults

		Blood collecting tube, 5 ml;<br>Total bilirubin;<br>Alkaline phosphatase reagents;<br>Electrolytes test;<br>BUN test;<br>Serum creatinine			
	**Breast Cancer**				
38	Basic palliative care for Breast Cancer: [29]	Ibuprofen, 400 mg tab;<br>Paracetamol, tablet, 500 mg;<br>Morphine injection (5mL);<br>Morphine oral liquid (10mg/mL);<br>Dexamethasone (4mg tablets);<br>Docusate (100mg tablet);<br>Senna, 7.5 mg tab;<br>Amitriptyline 25 mg tablets;<br>Loperamide 2 mg;<br>Metoclopramide (10mg tablet);<br>Morphine slow release tablet (10mg)	15 days	2 visits	1 visit from Pharmaceutical technician<br>15 visits with a nurse<br>1 long 2 hr visit from a specialist<br>15 visits with a GP

39	Diagnosis & treatment of breast cancer stages I and II [29]	X-ray film;<br>X-ray film chemistry;<br>Aspirating needle;<br>Aspirating syringe;<br>Syringe, needle + swab;<br>Specimen container;<br>Lidocaine HCl (in dextrose 7.5%), ampoule 2 ml;<br>Scalpel blade, disposable;<br>Biopsy needle;<br>Saline solution;<br>Formalin, 1 litre;<br>H and E staining;<br>Hormone receptor testing (immunoanalyzer);<br>Complete blood count;<br>Blood collecting tube, 5 ml;<br>Total bilirubin;<br>Alkaline phosphatase reagents;<br>Electrolytes test;<br>BUN test;<br>Serum creatinine;<br>Electrodes, electrocardiographic;<br>Gel, electrode;<br>Paper, Recording ECG;<br>Ultrasound gel;<br>Ultrasound probe cover;<br>Sponges;<br>Sutures;<br>Wound drainage kit;<br>Electrosurgical unit (monopolar pen, pad);<br>Skin preparatory solution, 500 ml vial;<br>Cefazolin, 1g;<br>Doxorubicin, 50 mg vial;<br>Doxorubicin, 10 mg vial;<br>Cyclophosphamide, 1 g;<br>Paclitaxel;<br>Cannulae;<br>Intravenous administration set;<br>Tamoxifen, 20 mg tablet;<br>Filgastrim;<br>Dexamethasone, 4 mg, 1 ampoule;<br>Ondansetron (4mg tablets);<br>Alendronate, 10 mg tab	8 days	2 visits	Nurses - 8 visits<br>Specialist Doctor - 8 visits<br>Laboratory technicians/assistants - 2 hrs<br>Assistant nurses and midwives 2 visits<br>Pharmaceutical technicians/assistants 2 visits<br>Other specialist doctors 2 visits<br>Radiographers/X-ray technicians 2 extra-long consults

40	Screening with mammography[29]	Mammography film;<br>Mammography film chemistry;<br>Ultrasound gel;<br>Ultrasound probe cover;<br>Scalpel blade, disposable;<br>Biopsy needle;<br>Saline solution;<br>Syringe, needle + swab;<br>Specimen container;<br>Lidocaine HCl (in dextrose 7.5%), ampoule 2 ml;<br>Wire localization needle;<br>Soft tissue marker/fiduciary clip;<br>Aspirating needle;<br>Aspirating syringe;<br>Formalin, 1 litre;<br>H and E staining		1 visit	Specialist doctor<br>Nurse<br>Generalist Doctor<br>X-Ray technician
