# Supplementary material for: Cost-Effectiveness of Population Level and Individual Level Interventions to Combat Non-communicable Disease in Eastern Sub-Saharan Africa and South East Asia: A WHO-CHOICE Analysis
Source: Int J Health Policy Manag. 2021 Jun 7;10(11):724–33. doi: 10.34172/ijhpm.2021.37 (PMC9278376; doi:10.34172/ijhpm.2021.37)
Supplement: Supplementary file 3 — Costing Inputs for Risk Factor Interventions. [file ijhpm-10-724-s003.pdf]

Article title: Cost-Effectiveness of Population Level and Individual Level Interventions to Combat Non-communicable Disease in Eastern Sub-Saharan Africa and South East Asia: A WHO-CHOICE Analysis

Journal name: International Journal of Health Policy and Management (IJHPM)

Authors' information: Melanie Y. Bertram[1]*, Daniel Chisholm[2], Rory Watts[1], Temo Waqanivalu[3], Vinayak Prasad[3], Cherian Varghese[4]

[1]Department of Health Systems Governance and Financing, World Health Organization, Geneva, Switzerland.

[2]Department of Mental Health and Substance Abuse, World Health Organization, Geneva, Switzerland.

[3]Department of Prevention of Non-Communicable Diseases, World Health Organization, Geneva, Switzerland.

[4]Department of Management of Non Communicable Diseases, Violence and Injury, World Health Organization, Geneva, Switzerland.

(*Corresponding author: bertramm@who.int)

Supplementary file 3. Costing Inputs for Risk Factor Interventions

	**Tobacco**	
41	Protect people from tobacco smoke[30]	This is a legislative intervention. Costs include human resource requirements, consultations, training, monitoring and evaluation.
42	Offer help to quit tobacco use[30]	Assumes that 50% of the population will see a GP each year, and that this will be equally representative of the proportion of the population who are tobacco users. For each individual the full cost of a GP visit is allocated to the brief intervention provided. This is likely an overestimate, thus the most conservative assumption.
43	Warn about the dangers of tobacco, 1[30]	This is a legislative intervention. Costs include human resource requirements, consultations, training, monitoring and evaluation.
44	Warn about the dangers of tobacco, 2, plain packaging[30]	This is a legislative intervention and requires passing of new legislation when compared to graphic warning labels. Costs include human resource requirements, consultations, training, monitoring and evaluation.
45	Enforce bans on tobacco advertising, promotion and sponsorship[30]	This is a legislative intervention and requires passing of new legislation when compared to graphic warning labels. Costs include human resource requirements, consultations, training, monitoring and evaluation.

46	Raise taxes on tobacco[30]	This is a legislative intervention and requires passing of new legislation when compared to graphic warning labels. Costs include human resource requirements, consultations, training, monitoring and evaluation.
47	Mass media campaigns for smoking prevention[30]	Generic advocacy/awareness campaigns are included as part of all health care interventions, with assumptions relying on information from the marketing literature and are outlined in the WHO CHOICE programme costing paper. We estimated that 10 times the intensity would be required to enact behaviour change in line with previous costing estimates 12
	**Alcohol**	
48	Increase in excise taxes on alcoholic beverages[8]	This is a legislative intervention. Costs include human resource requirements, consultations, training, monitoring and evaluation.
49	Enforcement of bans or comprehensive restrictions on exposure to alcohol advertising, promotion and sponsorship (across multiple types of media) [8]	This is a legislative intervention. Costs include human resource requirements, consultations, training, monitoring and evaluation.
50	Enforcement of restrictions on the physical availability of retailed alcohol (via reduced density of retail outlets and reduced hours of sale) [8]	This is a legislative intervention. Costs include human resource requirements, consultations, training, monitoring and evaluation.
51	Enforcement of drink-driving laws and blood alcohol concentration limits via sobriety checkpoints[8]	This is a legislative intervention. Costs include human resource requirements, consultations, training, monitoring and evaluation, as well as human resources for implementation.
52	Provision of brief psychosocial intervention for persons with hazardous and harmful alcohol use[8]	Assumes that 50% of the population will see a GP each year, and that this will be equally representative of the proportion of the population who consume alcohol at risky levels. For everyone the full cost of a GP visit is allocated to the brief intervention provided.
	**Physical Inactivity**	

53	Provide physical activity counselling as part of routine primary health care services through the use of a brief intervention [31]	Assumes that 50% of the population will see a GP each year, and that this will be equally representative of the proportion of the population who do not meet minimum physical activity requirements. For everyone the full cost of a GP visit is allocated to the brief intervention provided.
54	Implement community wide public education and awareness campaign for physical activity[31]	Generic advocacy/awareness campaigns are included as part of all health care interventions, with assumptions relying on information from the marketing literature and are outlined in the WHO CHOICE programme costing paper. We estimated that 10 times the intensity would be required to enact behaviour change in line with previous costing estimates 12
	**Unhealthy diet**	
55	Harness the Industry for voluntary reformulation [25]	Although not a legislative intervention we assume that costs include human resources consultations, training, monitoring and evaluation.
56	Adopt standards for front of pack labelling[25]	This is a legislative intervention. Costs include human resource requirements, consultations, training, monitoring and evaluation.
57	Knowledge: Education and communication[25]	Generic advocacy/awareness campaigns are included as part of all health care interventions, with assumptions relying on information from the marketing literature and are outlined in the WHO CHOICE programme costing paper. We estimated that 10 times the intensity would be required to enact behaviour change in line with previous costing estimates 12
58	Environment: Salt reduction strategies in community based eating spaces[25]	This is a legislative intervention. Costs include human resource requirements, consultations, training, monitoring and evaluation.
59	Trans fat elimination [25]	This is a legislative intervention. Costs include human resource requirements, consultations, training, monitoring and evaluation.
