# Supplementary material for: Cost-Effectiveness of Population Level and Individual Level Interventions to Combat Non-communicable Disease in Eastern Sub-Saharan Africa and South East Asia: A WHO-CHOICE Analysis
Source: Int J Health Policy Manag. 2021 Jun 7;10(11):724–33. doi: 10.34172/ijhpm.2021.37 (PMC9278376; doi:10.34172/ijhpm.2021.37)
Supplement: Supplementary file 4 — Costing Inputs for MNS Interventions. [file ijhpm-10-724-s004.pdf]

Article title: Cost-Effectiveness of Population Level and Individual Level Interventions to Combat Non-communicable Disease in Eastern Sub-Saharan Africa and South East Asia: A WHO-CHOICE Analysis

Journal name: International Journal of Health Policy and Management (IJHPM)

Authors' information: Melanie Y. Bertram[1]*, Daniel Chisholm[2], Rory Watts[1], Temo Waqanivalu[3], Vinayak Prasad[3], Cherian Varghese[4]

[1]Department of Health Systems Governance and Financing, World Health Organization, Geneva, Switzerland.

[2]Department of Mental Health and Substance Abuse, World Health Organization, Geneva, Switzerland.

[3]Department of Prevention of Non-Communicable Diseases, World Health Organization, Geneva, Switzerland.

[4]Department of Management of Non Communicable Diseases, Violence and Injury, World Health Organization, Geneva, Switzerland.

(*Corresponding author: bertramm@who.int)

Supplementary file 4. Costing Inputs for MNS Interventions

	**Anxiety**				
60	Basic psychosocial treatment for mild cases of anxiety disorder [8]			2 visits	1 nurse visit, 1 GP visit
61	Basic psychosocial and anti-depressant drug treatment for moderate-severe cases of anxiety disorder[8]	Fluoxetine, 20 mg tab;<br>Amitriptyline, 50 mg tab	2% of patients require 14 inpatient days	2 visits	1 nurse visit, 1 GP visit for psychosocial support<br>2 nurse visits, 2 GP visits for medication
62	Intensive psychosocial and anti-depressant drug treatment for moderate-severe cases of anxiety disorder[8]	Fluoxetine, 20 mg tab;<br>Amitriptyline, 50 mg tab	2% of patients require 14 inpatient days	2 visits for medication<br>10 visits for individual therapy (50% of patients)<br>8 visits for group therapy (50% of patients)	1 nurse visit, 1 GP visit for medication support<br>10 extra-long nurse visits for individual therapy for 50% of patients<br>8 peer group therapy sessions for 50% of patients
	**Depression**				

63	Basic psychosocial treatment for mild cases of depression[8]			2 visits	1 nurse visit, 1 GP visit
64	Basic psychosocial treatment and anti-depressant medication for first-episode moderate-severe cases of depression[8]	Fluoxetine, 20 mg tab;<br>Amitriptyline, 50 mg tab	2% of patients require 14 inpatient days	2 visits	1 nurse visit, 1 GP visit for psychosocial support<br>2 nurse visits, 2 GP visits for medication
65	Intensive psychosocial treatment and anti-depressant medication for first-episode moderate-severe cases of depression[8]	Fluoxetine, 20 mg tab;<br>Amitriptyline, 50 mg tab	2% of patients require 14 inpatient days	3 visits for medication<br>12 visits for individual therapy (50% of patients)<br>12 visits for group therapy (50% of patients)	1 nurse visit, 1 GP visit for medication support<br>12 extra-long nurse visits for individual therapy for 50% of patients<br>12 peer group therapy sessions for 50% of patients
66	Intensive psychosocial treatment and anti-depressant medication for recurrent moderate-severe cases of depression on an episodic basis[8]	Fluoxetine, 20 mg tab;<br>Amitriptyline, 50 mg tab	2% of patients require 14 inpatient days	3 visits for medication<br>12 visits for individual therapy (50% of patients)<br>12 visits for group therapy (50% of patients)	1 nurse visit, 1 GP visit for medication support<br>12 extra-long nurse visits for individual therapy for 50% of patients<br>12 peer group therapy sessions for 50% of patients
67	Intensive psychosocial treatment and anti-depressant medication for recurrent moderate-severe cases of depression on a maintenance basis[8]	Fluoxetine, 20 mg tab;<br>Amitriptyline, 50 mg tab	2% of patients require 14 inpatient days	3 visits for medication<br>12 visits for individual therapy (50% of patients)<br>12 visits for group therapy (50% of patients)	1 nurse visit, 1 GP visit for medication support<br>12 extra-long nurse visits for individual therapy for 50% of patients<br>12 peer group therapy sessions for 50% of patients
	**Bipolar disorder**				
68	Mood-Stabilizing Medication + Basic Psychosocial treatment for bipolar disorder (older drugs) [8]	Lithium, 300 mg;<br>Haloperidol, 5 mg tab;<br>Serum level test;<br>Thyroid function test;<br>Diazepam, 5 mg	15% of patients require 28 inpatient days<br><br>10% of patients require residential / long-term care (90 days on average)	6 visits	1 nurse visit, 1 GP visit for psychosocial support<br>2 nurse visits, 2 GP visits for medication

69	Mood-Stabilizing Medication + Intensive Psychosocial treatment for bipolar disorder (older drugs) [8]	Lithium, 300 mg;<br>Haloperidol, 5 mg tab;<br>Serum level test;<br>Thyroid function test;<br>Diazepam, 5 mg	15% of patients require 28 inpatient days<br><br>10% of patients require residential / long-term care (90 days on average)	6 visits for medication<br>12 visits for individual therapy (50% of patients)<br>12 visits for group therapy (50% of patients)	1 nurse visit, 1 GP visit for medication support<br>12 extra-long nurse visits for individual therapy for 50% of patients<br>12 peer group therapy sessions for 50% of patients
70	Mood-Stabilizing Medication + Basic Psychosocial treatment for bipolar disorder (newer drugs) [8]	Valproate, 500 mg;<br>Carbamazepine 200 mg;<br>Diazepam, 5 mg;<br>Serum level test;<br>Thyroid function test	15% of patients require 14 inpatient days<br><br>10% of patients require residential / long-term care (90 days on average)	6 visits	1 nurse visit, 1 GP visit for psychosocial support<br>2 nurse visits, 2 GP visits for medication
71	Mood-Stabilizing Medication + Intensive Psychosocial treatment for bipolar disorder (newer drugs) [8]	Valproate, 500 mg;<br>Carbamazepine 200 mg;<br>Diazepam, 5 mg;<br>Serum level test;<br>Thyroid function test	15% of patients require 28 inpatient days<br><br>10% of patients require residential / long-term care (90 days on average)	6 visits for medication<br>12 visits for individual therapy (50% of patients)<br>12 visits for group therapy (50% of patients)	1 nurse visit, 1 GP visit for medication support<br>12 extra-long nurse visits for individual therapy for 50% of patients<br>12 peer group therapy sessions for 50% of patients
	**Psychosis**				
72	Antipsychotic Medication + Basic Psychosocial treatment of psychosis (older drugs) [8]	Haloperidol, 5 mg tab;<br>Chlorpromazine, 100 mg;<br>Fluphenazine decanoate, 25 mg/ml;<br>EEG	15% of patients require 28 inpatient days<br><br>10% of patients require residential / long-term care (90 days on average)	2 visits	1 nurse visit, 1 GP visit for psychosocial support<br>2 nurse visits, 2 GP visits for medication
73	Antipsychotic Medication + Intensive Psychosocial treatment of psychosis (older drugs) [8]	Haloperidol, 5 mg tab;<br>Chlorpromazine, 100 mg;<br>Fluphenazine decanoate, 25 mg/ml;<br>EEG	15% of patients require 28 inpatient days<br><br>10% of patients require residential / long-term care (90 days on average)	6 visits for medication<br>12 visits for individual therapy (50% of patients)<br>12 visits for group therapy (50% of patients)	1 nurse visit, 1 GP visit for medication support<br>12 extra-long nurse visits for individual therapy for 50% of patients<br>12 peer group therapy sessions for 50% of patients
74	Antipsychotic Medication + Basic Psychosocial treatment	Risperidone, 2 mg tab;<br>Biperiden, 2 mg tab;<br>EEG	15% of patients require 28 inpatient days	6 visits	1 nurse visit, 1 GP visit for psychosocial support<br>2 nurse visits, 2 GP visits for medication

	of psychosis (newer drugs) [8]		10% of patients require residential / long-term care (90 days on average)		
75	Antipsychotic Medication + Intensive Psychosocial treatment of psychosis (newer drugs) [8]	Risperidone, 2 mg tab;<br>Biperiden, 2 mg tab;<br>EEG	15% of patients require 28 inpatient days<br><br>10% of patients require residential / long-term care (90 days on average)	6 visits for medication<br>12 visits for individual therapy (50% of patients)<br>12 visits for group therapy (50% of patients)	1 nurse visit, 1 GP visit for medication support<br>12 extra-long nurse visits for individual therapy for 50% of patients<br>12 peer group therapy sessions for 50% of patients
	**Epilepsy**				
76	Antiepileptic Medication + Basic Psychosocial treatment of epilepsy (older drugs) [8]	Phenobarbital, 100 mg;<br>Phenytoin, 100 mg;		4 visits	1 nurse visit, 1 GP visit for psychosocial support<br>2 nurse visits, 2 GP visits for medication
77	Antiepileptic Medication + Basic Psychosocial treatment of epilepsy (newer drugs) [8]	Carbamazepine 200 mg		4 visits	1 nurse visit, 1 GP visit for psychosocial support<br>2 nurse visits, 2 GP visits for medication
