# Supplementary material for: Cost-Effectiveness of Population Level and Individual Level Interventions to Combat Non-communicable Disease in Eastern Sub-Saharan Africa and South East Asia: A WHO-CHOICE Analysis
Source: Int J Health Policy Manag. 2021 Jun 7;10(11):724–33. doi: 10.34172/ijhpm.2021.37 (PMC9278376; doi:10.34172/ijhpm.2021.37)
Supplement: Supplementary file 5 — Cost, Impact and ACER Values for All Interventions. [file ijhpm-10-724-s005.pdf]

Article title: Cost-Effectiveness of Population Level and Individual Level Interventions to Combat Non-communicable Disease in Eastern Sub-Saharan Africa and South East Asia: A WHO-CHOICE Analysis

Journal name: International Journal of Health Policy and Management (IJHPM)

Authors' information: Melanie Y. Bertram[1]*, Daniel Chisholm[2], Rory Watts[1], Temo Waqanivalu[3], Vinayak Prasad[3], Cherian Varghese[4]

[1]Department of Health Systems Governance and Financing, World Health Organization, Geneva, Switzerland.

[2]Department of Mental Health and Substance Abuse, World Health Organization, Geneva, Switzerland.

[3]Department of Prevention of Non-Communicable Diseases, World Health Organization, Geneva, Switzerland.

[4]Department of Management of Non Communicable Diseases, Violence and Injury, World Health Organization, Geneva, Switzerland.

(*Corresponding author: bertramm@who.int)

Supplementary file 5. Cost, Impact and ACER Values for All Interventions

Intervention code	Coverage	Name	South East Asia			Eastern Sub-Saharan Africa		
**Cardiovascular Disease**			**Cost per 10 million**	**HLY per 10 million (no disc)**	**ACER**	**Cost per 10 million**	**HLY per 10 million (no disc)**	**ACER**
CVD-1	50	Prevention: Comb. Tx for total CVD risk > 30%, 50% coverage	$265,229,707	9,039,000	$29	$504,982,376	19,044,395	$27
CVD-2	50	Prevention: Tx SBP > 160 mmHG, total CVD risk < 30%, 50% coverage	$281,056,741	1,598,781	$176	$540,172,822	6,384,497	$85
CVD-3	50	Prevention: Tx SBP > 140 mmHG, total CVD risk < 30%, 50% coverage	$181,292,781	3,445,038	$53	$381,881,749	7,642,586	$50
CVD-4	50	Prevention: Tx Chol > 8mmol/L, total CVD risk < 30%, 50% coverage	$51,509,752	26,783	$1,923	$169,326,999	4,289	$39,476
CVD-5	50	Prevention: Tx Chol > 6mmol/L, total CVD risk < 30%, 50% coverage	$403,631,938	2,397,396	$168	$724,262,599	1,620,356	$447

CVD-6	50	Prevention: Comb. Tx for total CVD risk > 20%, 50% coverage	$486,428,986	15,415,467	$32	$854,159,048	33,130,080	$26
CVD-7	50	Prevention: Tx SBP > 160 mmHG, total CVD risk < 20%, 50% coverage	$185,083,878	1,075,498	$172	$387,896,915	5,011,649	$77
CVD-8	50	Prevention: Tx SBP > 140 mmHG, total CVD risk < 20%, 50% coverage	$771,029,755	3,039,792	$254	$1,317,591,380	7,101,904	$186
CVD-9	50	Prevention: Tx Chol > 8mmol/L, total CVD risk < 20%, 50% coverage	$50,740,795	19,157	$2,649	$168,115,143	106,551	$1,578
CVD-10	50	Prevention: Tx Chol > 6mmol/L, total CVD risk < 20%, 50% coverage	$321,950,196	2,117,076	$152	$595,534,254	1,503,991	$396
CVD-11	50	Treatment: Aspirin, 50% coverage	$10,156,351	106,882	$95	$25,423,243	97,205	$262
CVD-12	50	Treatment: Treat post acute IHD combination, 50% coverage	$609,165,471	596,465	$1,021	$1,048,375,366	913,936	$1,147
CVD-13	50	Treatment: Treat post acute Stroke combination, 50% coverage	$565,589,900	544,109	$1,039	$935,620,079	657,824	$1,422
CVD-14	80	Prevention: Comb. Tx for total CVD risk > 30%, 80% coverage	$399,748,409	12,543,005	$32	$717,321,525	29,025,429	$25
CVD-15	80	Prevention: Tx SBP > 160 mmHG, total CVD risk < 30%, 80% coverage	$419,769,195	2,569,709	$163	$760,261,751	10,285,950	$74
CVD-16	80	Prevention: Tx SBP > 140 mmHG, total CVD risk < 30%, 80% coverage	$260,781,061	5,675,619	$46	$508,002,295	12,324,030	$41
CVD-17	80	Prevention: Tx Chol > 8mmol/L, total CVD risk < 30%, 80% coverage	$52,639,019	42,857	$1,228	$171,106,696	6,863	$24,932
CVD-18	80	Tx Chol > 6mmol/L, total CVD risk < 30%, 80% coverage	$618,088,725	3,683,730	$168	$1,062,241,034	2,598,182	$409
CVD-19	80	Comb. Tx for total CVD risk > 20%, 80% coverage	$765,489,465	19,914,191	$38	$1,294,707,754	41,750,500	$31
CVD-20	80	Tx SBP > 160 mmHG, total CVD risk < 20%, 80% coverage	$265,186,179	1,726,042	$154	$514,991,701	8,058,720	$64
CVD-21	80	Tx SBP > 140 mmHG, total CVD risk < 20%, 80% coverage	$1,208,247,262	4,896,924	$247	$2,011,305,106	11,442,499	$176
CVD-22	80	Tx Chol > 8mmol/L, total CVD risk < 20%, 80% coverage	$51,408,499	30,654	$1,677	$169,167,428	152,740	$1,108
CVD-23	80	Tx Chol > 6mmol/L, total CVD risk < 20%, 80% coverage	$486,816,276	3,248,968	$150	$855,358,997	2,410,987	$355

CVD-24	80	Aspirin, 80% coverage	$12,144,170	165,527	$73	$30,016,321	150,590	$199
CVD-25	80	Treat post acute IHD combination, 80% coverage	$989,283,022	1,004,451	$985	$1,649,151,246	1,597,520	$1,032
CVD-26	80	Treat post acute Stroke combination, 80% coverage	$872,464,097	1,011,651	$862	$1,389,889,834	1,165,001	$1,193
CVD-27	95	Comb. Tx for total CVD risk > 30%, 95% coverage	$467,545,191	13,915,963	$34	$824,339,440	31,339,161	$26
CVD-28	95	Tx SBP > 160 mmHG, total CVD risk < 30%, 95% coverage	$495,779,071	3,132,179	$158	$880,863,267	12,256,999	$72
CVD-29	95	Tx SBP > 140 mmHG, total CVD risk < 30%, 95% coverage	$300,806,241	6,625,521	$45	$571,508,482	14,692,383	$39
CVD-30	95	Tx Chol > 8mmol/L, total CVD risk < 30%, 95% coverage	$53,203,740	50,895	$1,045	$171,996,681	8,150	$21,104
CVD-31	95	Tx Chol > 6mmol/L, total CVD risk < 30%, 95% coverage	$725,679,448	4,042,845	$179	$1,231,801,274	3,088,687	$399
CVD-32	95	Comb. Tx for total CVD risk > 20%, 95% coverage	$905,067,222	21,762,893	$42	$1,515,057,123	44,905,380	$34
CVD-33	95	Tx SBP > 160 mmHG, total CVD risk < 20%, 95% coverage	$305,427,904	2,052,804	$149	$578,841,470	9,593,697	$60
CVD-34	95	Tx SBP > 140 mmHG, total CVD risk < 20%, 95% coverage	$1,428,642,973	5,835,032	$245	$2,360,997,254	13,635,625	$173
CVD-35	95	Tx Chol > 8mmol/L, total CVD risk < 20%, 95% coverage	$51,742,385	36,403	$1,421	$169,693,623	197,416	$860
CVD-36	95	Tx Chol > 6mmol/L, total CVD risk < 20%, 95% coverage	$569,432,231	3,671,065	$155	$985,559,639	2,865,831	$344
CVD-37	95	Aspirin, 95% coverage	$12,865,587	173,574	$74	$32,313,063	175,840	$184
CVD-38	95	Treat post acute IHD combination, 95% coverage	$1,193,436,413	1,224,876	$974	$1,971,815,731	1,988,804	$991
CVD-39	95	Treat post acute Stroke combination, 95% coverage	$1,026,389,343	1,147,591	$894	$1,617,747,323	1,461,416	$1,107
CVD-40	95	prevention - 20% risk plus 160mmHG	$1,244,977,332	23,815,697	$52	$2,080,319,886	41,048,900	$51
CVD-41	95	prevention - 20% risk plus 140 mmHg	$2,368,192,401	27,597,925	$86	$3,862,475,670	47,567,975	$81
CVD-42	95	prevention - 20%risk 8mmol/L	$936,592,274	21,799,296	$43	$1,574,238,159	37,573,417	$42
CVD-43	95	prevention - 20%risk + 6mmol/L	$1,454,282,120	25,433,958	$57	$2,390,104,174	43,838,146	$55
CVD-44	95	prevention- 20% risk + 8mmol/L+160mmHg	$1,255,497,253	23,852,099	$53	$2,107,748,181	41,111,644	$51
CVD-45	95	prevention- 20% risk + 6mmol/L+160mmHg	$1,805,189,585	27,486,761	$66	$2,979,920,747	53,431,842	$56
CVD-46	95	prevention- 20% risk + 6mmol/L+140mmHg	$2,928,404,655	31,268,990	$94	$4,762,076,531	57,473,770	$83

CVD-47	95	prevention package plus treat stroke	$4,253,285,116	32,416,581	$131	$6,428,186,686	58,935,186	$109
CVD-48	95	prevention package plus treat stroke plus treat ihd	$4,623,489,723	33,641,457	$137	$7,455,878,755	60,923,990	$122
CVD-49	95	prevention package plus treat stroke plus treat ihd plus aspirin	$6,295,933,903	33,815,031	$186	$9,515,687,046	61,099,830	$156
**Diabetes Mellitus**								
DM-1	50	Standard Glycemic control, 50% coverage	$1,121,365,306	162,226	$6,912	$850,060,574	132,875	$6,397
DM-2	50	Intensive Glycemic control, 50% coverage	$943,176,721	87,511	$10,778	$748,447,962	69,631	$10,749
DM-3	50	Retinopathy Screening + photocoagulation, 50% coverage	$388,972,419	60,406	$6,439	$401,469,841	44,734	$8,975
DM-4	50	Neuropathy screening and preventive foot care, 50% coverage	$78,525,814	106,059	$740	$161,881,836	78,443	$2,064
DM-5	50	Standard plus intensive glycemic control, 50% coverage	$2,041,102,879	253,253	$8,060	$1,476,826,872	203,219	$7,267
DM-6	50	standard GC plus retinopathy screening, 50% coverage	$1,968,542,192	225,145	$8,743	$1,494,383,639	177,939	$8,398
DM-7	50	standard GC plus neuropathy screening, 50% coverage	$1,273,361,231	271,769	$4,685	$926,142,030	211,857	$4,372
DM-8	50	intensive GC plus retinopathy screening, 50% coverage	$1,725,523,389	150,673	$11,452	$1,347,317,769	114,663	$11,750
DM-9	50	intensive GC plus neuropathy screening, 50% coverage	$1,089,534,294	197,264	$5,523	$823,300,808	148,557	$5,542
DM-10	80	Standard Glycemic control, 80% coverage	$1,781,996,807	261,593	$6,812	$1,276,856,728	212,909	$5,997
DM-11	80	Intensive Glycemic control, 80% coverage	$1,490,212,583	140,586	$10,600	$1,108,541,999	110,460	$10,036
DM-12	80	Retinopathy Screening + photocoagulation, 80% coverage	$590,537,938	96,904	$6,094	$509,691,653	70,869	$7,192
DM-13	80	Neuropathy screening and preventive foot care, 80% coverage	$98,955,070	170,481	$580	$166,950,927	124,520	$1,341
DM-14	80	Standard plus intensive glycemic control, 80% coverage	$3,255,125,231	410,086	$7,938	$2,265,718,232	328,213	$6,903
DM-15	80	standard GC plus retinopathy screening, 80% coverage	$3,130,129,024	364,030	$8,599	$2,246,680,061	287,033	$7,827
DM-16	80	standard GC plus neuropathy screening, 80% coverage	$2,019,980,293	440,307	$4,588	$1,381,603,994	342,267	$4,037
DM-17	80	intensive GC plus retinopathy screening, 80% coverage	$2,731,269,471	242,709	$11,253	$2,001,974,515	184,414	$10,856

DM-18	80	intensive GC plus neuropathy screening, 80% coverage	$1,718,270,594	318,402	$5,397	$1,210,484,636	239,292	$5,059
DM-19	95	Standard Glycemic control, 95% coverage	$2,115,845,320	311,861	$6,785	$1,492,663,488	253,611	$5,886
DM-20	95	Intensive Glycemic control, 95% coverage	$1,765,246,700	167,286	$10,552	$1,290,834,202	131,378	$9,825
DM-21	95	Retinopathy Screening + photocoagulation, 95% coverage	$692,400,936	115,225	$6,009	$564,402,850	84,241	$6,700
DM-22	95	Neuropathy screening and preventive foot care, 95% coverage	$109,233,939	202,916	$538	$169,517,246	148,129	$1,144
DM-23	95	Standard plus intensive glycemic control, 95% coverage	$3,867,202,969	489,923	$7,893	$2,664,830,255	391,596	$6,805
DM-24	95	standard GC plus retinopathy screening, 95% coverage	$3,717,136,445	434,572	$8,554	$2,627,063,485	342,252	$7,676
DM-25	95	standard GC plus neuropathy screening, 95% coverage	$2,397,237,404	526,170	$4,556	$1,611,908,604	408,427	$3,947
DM-26	95	intensive GC plus retinopathy screening, 95% coverage	$3,237,451,650	289,195	$11,195	$2,333,310,197	219,561	$10,627
DM-27	95	intensive GC plus neuropathy screening, 95% coverage	$2,034,388,993	379,772	$5,357	$1,406,490,807	285,118	$4,933
**Asthma**								
AST-1	50	Inhaled short acting beta agonist for intermittent asthma, 50% coverage	$10,452,854	2,663	$3,926	$26,069,059	11,677	$2,232
AST-2	50	Low dose inhaled beclometasone + SABA, 50% coverage	$20,750,654	44,216	$469	$27,383,991	193,917	$141
AST-3	50	High dose inhaled beclometasone +SABA, 50% coverage	$19,948,358	56,145	$355	$27,320,598	246,230	$111
AST-4	50	Theophylline + High dose inhaled beclometasone +SABA, 50% coverage	$11,399,232	50,214	$227	$26,965,313	220,222	$122
AST-5	50	Oral Prednisolone + Theophylline + High dose inhaled beclometasone +SABA, 50% coverage	$22,255,171	77,401	$288	$30,307,804	339,455	$89
AST-6	80	Inhaled short acting beta agonist for intermittent asthma, 50% coverage	$13,294,744	4,260	$3,121	$31,048,809	18,684	$1,662
AST-7	80	Low dose inhaled beclometasone + SABA, 80% coverage	$28,765,939	70,746	$407	$33,152,701	310,268	$107

AST-8	80	High dose inhaled beclometasone +SABA, 80% coverage	$27,482,266	89,831	$306	$33,051,273	393,969	$84
AST-9	80	Theophylline + High dose inhaled beclometasone +SABA, 80% coverage	$15,342,304	80,343	$191	$32,482,815	352,355	$92
AST-10	80	Oral Prednisolone + Theophylline + High dose inhaled beclometasone +SABA, 80% coverage	$32,746,311	123,842	$264	$37,830,801	543,128	$70
AST-11	95	Inhaled short acting beta agonist for intermittent asthma, 95% coverage	$14,715,689	5,059	$2,909	$33,538,684	22,187	$1,512
AST-12	95	Low dose inhaled beclometasone + SABA, 95% coverage	$32,773,582	84,011	$390	$36,037,056	368,443	$98
AST-13	95	High dose inhaled beclometasone +SABA, 95% coverage	$31,249,219	106,675	$293	$35,916,610	467,838	$77
AST-14	95	Theophylline + High dose inhaled beclometasone +SABA, 95% coverage	$17,313,841	95,407	$181	$35,241,567	418,421	$84
AST-15	95	Oral Prednisolone + Theophylline + High dose inhaled beclometasone +SABA, 95% coverage	$37,991,881	147,063	$258	$41,592,299	644,965	$64
**COPD**								
COP-1	50	Smoking cessation, 50% coverage	$75,840,651	552,424	$137	$278,136,851	1,066,922	$261
COP-2	50	Inhaled salbutamol, 50% coverage	$261,264,309	1,190,912	$219	$484,599,508	3,447,415	$141
COP-3	50	Low-dose oral theophylline, 50% coverage	$187,895,877	442,615	$425	$368,198,540	1,286,991	$286
COP-4	50	Ipratropium inhaler, 50% coverage	$252,893,070	384,124	$658	$472,169,552	1,111,126	$425
COP-5	50	Antibiotics, 50% coverage	$133,089,967	329,740	$404	$408,215,253	446,992	$913
COP-6	50	Oral prednisolone, 50% coverage	$110,327,936	326,650	$338	$349,527,204	945,577	$370
COP-7	50	Oxygen, concentration 24-28%, 50% coverage	$158,423,330	621,307	$255	$46,558,973	1,505,191	$31
COP-8	50	Salbutamol plus theophylline, 50% coverage	$405,189,655	1,636,224	$248	$716,356,821	4,734,444	$151
COP-9	50	Salbutamol plus theophylline plus inpratropium, 50% coverage	$628,394,521	2,020,348	$311	$1,073,095,363	5,845,570	$184
COP-10	50	Antibiotics plus prednisolone plus oxygen, 50% coverage	$1,706,318,465	1,287,225	$1,326	$804,301,430	2,652,683	$303

COP-11	80	Smoking cessation, 80% coverage	$80,837,974	887,715	$91	$310,314,586	1,695,317	$183
COP-12	80	Inhaled salbutamol, 80% coverage	$386,170,122	1,905,756	$203	$694,526,896	5,504,677	$126
COP-13	80	Low-dose oral theophylline, 80% coverage	$269,572,623	708,508	$380	$509,982,278	2,054,988	$248
COP-14	80	Ipratropium inhaler, 80% coverage	$375,383,344	613,682	$612	$679,886,296	1,777,802	$382
COP-15	80	Antibiotics, 80% coverage	$166,385,307	530,459	$314	$500,460,905	704,547	$710
COP-16	80	Oral prednisolone, 80% coverage	$130,216,116	522,640	$249	$412,365,877	1,509,854	$273
COP-17	80	Oxygen, concentration 24-28%, 80% coverage	$249,015,073	996,188	$250	$63,811,361	2,399,275	$27
COP-18	80	Salbutamol plus theophylline, 80% coverage	$610,453,374	2,617,958	$233	$1,052,964,247	7,575,110	$139
COP-19	80	Salbutamol plus theophylline plus inpratropium, 80% coverage	$961,931,341	3,232,556	$298	$1,611,919,357	9,352,913	$172
COP-20	80	Antibiotics plus prednisolone plus oxygen, 80% coverage	$2,604,411,376	2,071,745	$1,257	$976,638,144	4,222,763	$231
COP-21	95	Smoking cessation, 95% coverage	$80,905,209	1,057,007	$77	$321,325,179	2,015,998	$159
COP-22	95	Inhaled salbutamol, 95% coverage	$446,907,485	2,263,086	$197	$796,734,339	6,536,804	$122
COP-23	95	Low-dose oral theophylline, 95% coverage	$308,673,524	841,108	$367	$578,079,260	2,440,299	$237
COP-24	95	Ipratropium inhaler, 95% coverage	$434,873,023	728,748	$597	$780,895,160	2,111,140	$370
COP-25	95	Antibiotics, 95% coverage	$180,292,966	631,646	$285	$540,938,232	838,371	$645
COP-26	95	Oral prednisolone, 95% coverage	$137,403,588	620,732	$221	$438,290,448	1,792,952	$244
COP-27	95	Oxygen, concentration 24-28%, 95% coverage	$294,434,148	1,184,695	$249	$72,461,842	2,851,059	$25
COP-28	95	Salbutamol plus theophylline, 95% coverage	$711,023,830	3,108,825	$229	$1,217,966,473	8,995,444	$135
COP-29	95	Salbutamol plus theophylline plus inpratropium, 95% coverage	$1,126,183,899	3,838,661	$293	$1,877,313,444	11,106,584	$169
COP-30	95	Antibiotics plus prednisolone plus oxygen, 95% coverage	$634,887,004	2,467,552	$257	$1,051,690,522	5,017,981	$210
**Anxiety**								
ANX-1	50	Basic psychosocial treatment for mild cases of anxiety disorder	$247,108,300	166,339	$1,486	$424,348,592	436,738	$972
ANX-2	50	Basic psychosocial and anti-depressant drug treatment for moderate-severe cases of anxiety disorder	$267,669,821	165,748	$1,615	$426,853,199	458,476	$931

ANX-3	50	Intensive psychosocial and anti-depressant drug treatment for moderate-severe cases of anxiety disorder	$501,104,158	208,307	$2,406	$592,104,041	591,536	$1,001
**Depression**								
DEP-1	50	Basic psychosocial treatment for mild cases of depression	$326,958,789	664,717	$492	$448,801,495	1,700,970	$264
DEP-2	50	Basic psychosocial treatment and anti-depressant medication for first-episode moderate-severe cases of depression	$167,609,365	434,860	$385	$284,765,188	1,114,547	$255
DEP-3	50	Intensive psychosocial treatment and anti-depressant medication for first-episode moderate-severe cases of depression	$291,720,721	548,131	$532	$353,683,218	1,412,495	$250
DEP-4	50	Intensive psychosocial treatment and anti-depressant medication for recurrent moderate-severe cases of depression on an episodic basis	$574,472,702	1,252,653	$459	$549,751,103	3,229,200	$170
DEP-5	50	Intensive psychosocial treatment and anti-depressant medication for recurrent moderate-severe cases of depression on a maintenance basis	$660,822,502	2,234,969	$296	$634,697,669	5,694,731	$111
**Bipolar Disorder**								
BIP-1	50	Mood-Stabilizing Medication + Basic Psychosocial treatment for bipolar disorder (older drugs)						
BIP-2	50	Mood-Stabilizing Medication + Intensive Psychosocial treatment for bipolar disorder (older drugs)						
BIP-3	50	Mood-Stabilizing Medication + Basic Psychosocial treatment for bipolar disorder (newer drugs)						
BIP-4	50	Mood-Stabilizing Medication + Intensive Psychosocial treatment for bipolar disorder (newer drugs)						
BIP-5	80	Mood-Stabilizing Medication + Basic Psychosocial treatment for bipolar disorder (older drugs)	$767,621,808	511,304	$1,501	$985,331,403	1,130,380	$872
BIP-6	80	Mood-Stabilizing Medication + Intensive Psychosocial treatment for bipolar disorder (older drugs)	$1,214,691,346	596,521	$2,036	$1,169,053,899	1,318,776	$886
BIP-7	80	Mood-Stabilizing Medication + Basic Psychosocial treatment for bipolar disorder (newer drugs)	$1,188,048,038	511,304	$2,324	$1,594,178,590	1,130,380	$1,410

BIP-8	80	Mood-Stabilizing Medication + Intensive Psychosocial treatment for bipolar disorder (newer drugs)	$1,647,046,244	596,521	$2,761	$1,783,399,745	1,318,776	$1,352
BIP-9	95	Mood-Stabilizing Medication + Basic Psychosocial treatment for bipolar disorder (older drugs)	$903,882,378	607,174	$1,489	$1,139,888,502	1,342,326	$849
BIP-10	95	Mood-Stabilizing Medication + Intensive Psychosocial treatment for bipolar disorder (older drugs)	$1,434,777,455	708,369	$2,025	$1,358,058,966	1,566,047	$867
BIP-11	95	Mood-Stabilizing Medication + Basic Psychosocial treatment for bipolar disorder (newer drugs)	$1,403,138,526	607,174	$2,311	$1,862,894,537	1,342,326	$1,388
BIP-12	95	Mood-Stabilizing Medication + Intensive Psychosocial treatment for bipolar disorder (newer drugs)	$1,948,142,951	708,369	$2,750	$2,087,501,280	1,566,047	$1,333
**Psycosis**								
PSY-1	50	AntiPsychotic Medication + Basic Psychosocial treatment of psychosis (older drugs)	$275,439,477	248,176	$1,110	$393,248,399	463,092	$849
PSY-2	50	AntiPsychotic Medication + Intensive Psychosocial reatment of psychosis (older drugs)	$314,318,864	421,899	$745	$386,643,974	787,256	$491
PSY-3	50	AntiPsychotic Medication + Basic Psychosocial treatment of psychosis (newer drugs)	$294,827,241	248,176	$1,188	$416,706,903	463,092	$900
PSY-4	50	AntiPsychotic Medication + Intensive Psychosocial treatment of psychosis (newer drugs)	$334,187,793	421,899	$792	$410,540,276	787,256	$521
PSY-5	80	AntiPsychotic Medication + Basic Psychosocial treatment of psychosis (older drugs)	$414,685,934	397,081	$1,044	$532,274,598	740,947	$718
PSY-6	80	AntiPsychotic Medication + Intensive Psychosocial reatment of psychosis (older drugs)	$478,906,130	675,039	$709	$518,518,216	1,259,610	$412
PSY-7	80	AntiPsychotic Medication + Basic Psychosocial treatment of psychosis (newer drugs)	$445,598,112	397,081	$1,122	$569,808,205	740,947	$769
PSY-8	80	AntiPsychotic Medication + Intensive Psychosocial treatment of psychosis (newer drugs)	$510,807,736	675,039	$757	$556,527,565	1,259,610	$442
PSY-9	95	AntiPsychotic Medication + Basic Psychosocial treatment of psychosis (older drugs)						

PSY-10	95	AntiPsychotic Medication + Intensive Psychosocial reatment of psychosis (older drugs)						
PSY-11	95	AntiPsychotic Medication + Basic Psychosocial treatment of psychosis (newer drugs)						
PSY-12	95	AntiPsychotic Medication + Intensive Psychosocial treatment of psychosis (newer drugs)						
**Epilepsy**								
EPI-1	50	AntiEpileptic Medication + Basic Psychosocial treatment of epilepsy (older drugs)	$125,319,290	1,132,332	$111	$230,568,993	3,038,777	$76
EPI-2	50	AntiEpileptic Medication + Basic Psychosocial treatment of epilepsy (newer drugs)	$152,541,478	1,132,332	$135	$293,262,649	3,038,777	$97
EPI-3	80	AntiEpileptic Medication + Basic Psychosocial treatment of epilepsy (older drugs)	$164,273,558	1,644,673	$100	$264,672,731	4,544,746	$58
EPI-4	80	AntiEpileptic Medication + Basic Psychosocial treatment of epilepsy (newer drugs)	$204,028,333	1,644,673	$124	$358,740,667	4,544,746	$79
EPI-5	95	AntiEpileptic Medication + Basic Psychosocial treatment of epilepsy (older drugs)						
EPI-6	95	AntiEpileptic Medication + Basic Psychosocial treatment of epilepsy (newer drugs)						
**Risk Factor Prevention**								
PA-1		Brief Intervention - Physical Activity	$65,588,146	273,571	$240	$29,389,639	396,742	$74
UD-1		Adopt interpretive front-of-pack nutrient labelling systems	$14,200,394	3,235,397	$4	$42,251,147	6,890,161	$6
UD-2		Set target levels for the amount of salt in food and implement strategies to promote reformulation	$470,988	2,077,102	$0	$1,474,691	4,358,992	$0
UD-3		Implement multicomponent salt reduction strategies in community settings including schools, workplaces and hospitals	$4,246,565	804,444	$5	$17,194,933	955,082	$18
UD-4		Complete Elimination of Industrial Transfats	$148,541,225	25,398	$5,849	$186,877,181	36,108	$5,176
TOB-1		Protect - Smoke Free Policies	$14,200,394	252,101	$56	$9,792,328	741,132	$13
TOB-2		Offer to Help Quit (Brief Intervention) Tobacco	$65,588,146	125,948	$521	$29,389,639	370,302	$79
TOB -3		Warning Labels Tobacco	$6,678,474	252,101	$26	$4,384,947	741,132	$6

TOB-4		Enforce Advertising Bans Tobacco	$7,086,916	631,802	$11	$5,021,851	1,856,812	$3
TOB-5		Raise Taxes Tobacco	$5,400,016	13,460,010	$0	$4,908,197	34,757,605	$0
**Cancers**		0						
CVC-1		Basic palliative care for Cervical cancer: home-based and hospital care with multi-disciplinary team and access to opiates and essential supportive medicines	$211,330,021	2,769	$76,313	$155,645,256	25,520	$6,099
CVC-2		Basic palliative care for Cervical cancer: home-based and hospital care with multi-disciplinary team and access to opiates and essential supportive medicines	$234,013,422	4,431	$52,815	$192,347,733	40,832	$4,711
CVC-3		Basic palliative care for Cervical cancer: home-based and hospital care with multi-disciplinary team and access to opiates and essential supportive medicines	$245,355,123	5,262	$46,632	$169,911,240	48,488	$3,504
CVC-4		Diagnosis & treatment of cervical cancer stages I and II (using surgery, radiotherapy and chemotherapy as needed)	$197,825,685	171,314	$1,155	$173,358,489	918,353	$189
CVC-5		Diagnosis & treatment of cervical cancer stages I and II (using surgery, radiotherapy and chemotherapy as needed)	$220,224,197	335,061	$657	$223,364,806	1,777,983	$126
CVC-6		Diagnosis & treatment of cervical cancer stages I and II (using surgery, radiotherapy and chemotherapy as needed)	$231,423,452	445,670	$519	$460,559,737	2,355,450	$196
CVC-7		HPV vaccination (2 doses) for preventing cervical cancer	$232,111,233	1,112,285	$209	$286,113,995	5,215,136	$55
CVC-8		HPV vaccination (2 doses) for preventing cervical cancer	$262,249,565	1,499,743	$175	$373,016,469	6,773,262	$55
CVC-9		HPV vaccination (2 doses) for preventing cervical cancer	$277,318,731	1,630,353	$170	$233,063,199	7,297,912	$32
CVC-10		HPV vaccination (2 doses) for preventing cervical cancer & Prevention of cervical cancer through screening with HPV test: women 30-49 yrs every 3 years linked with timely treatment of pre-cancerous lesions	$429,588,735	4,284,936	$100	$763,994,050	24,649,274	$31

CVC-11		HPV vaccination (2 doses) for preventing cervical cancer & Prevention of cervical cancer through screening with HPV test: women 30-49 yrs every 3 years linked with timely treatment of pre-cancerous lesions	$576,910,674	4,927,198	$117	$1,143,584,301	29,121,530	$39
CVC-12		HPV vaccination (2 doses) for preventing cervical cancer & Prevention of cervical cancer through screening with HPV test: women 30-49 yrs every 3 years linked with timely treatment of pre-cancerous lesions	$650,237,299	5,109,215	$127	$1,567,691,503	30,413,350	$52
CVC-13		HPV vaccination (2 doses) for preventing cervical cancer & Prevention of cervical cancer through screening with PAP: women 30-49 yrs every 3 years linked with timely treatment of pre-cancerous lesions	$724,071,165	4,472,666	$162	$1,384,388,264	25,894,136	$53
CVC-14		HPV vaccination (2 doses) for preventing cervical cancer & Prevention of cervical cancer through screening with PAP: women 30-49 yrs every 3 years linked with timely treatment of pre-cancerous lesions	$1,028,175,176	5,059,125	$203	$2,118,330,191	30,073,810	$70
CVC-15		HPV vaccination (2 doses) for preventing cervical cancer & Prevention of cervical cancer through screening with PAP: women 30-49 yrs every 3 years linked with timely treatment of pre-cancerous lesions	$1,179,834,841	5,222,303	$226	$2,459,686,951	31,251,433	$79
CVC-16		HPV vaccination (2 doses) for preventing cervical cancer & Prevention of cervical cancer through screening with VIA: women 30-49 yrs every 3 years linked with timely treatment of pre-cancerous lesions	$547,369,903	4,541,842	$121	$978,065,571	26,362,292	$37
CVC-17		HPV vaccination (2 doses) for preventing cervical cancer &	$759,706,652	5,106,391	$149	$1,489,562,570	30,421,065	$49

		Prevention of cervical cancer through screening with VIA: women 30-49 yrs every 3 years linked with timely treatment of pre-cancerous lesions						
CVC-18		HPV vaccination (2 doses) for preventing cervical cancer & Prevention of cervical cancer through screening with VIA: women 30-49 yrs every 3 years linked with timely treatment of pre-cancerous lesions	$865,736,098	5,262,580	$165	$2,668,626,960	31,554,286	$85
CVC-19		Prevention of cervical cancer through screening with HPV test: women 30-49 yrs every 3 years linked with timely treatment of pre-cancerous lesions	$552,596,879	3,776,827	$146	$1,217,328,618	21,058,982	$58
CVC-20		Prevention of cervical cancer through screening with HPV test: women 30-49 yrs every 3 years linked with timely treatment of pre-cancerous lesions	$744,126,041	4,384,869	$170	$1,801,182,813	25,096,943	$72
CVC-21		Prevention of cervical cancer through screening with HPV test: women 30-49 yrs every 3 years linked with timely treatment of pre-cancerous lesions	$839,265,280	4,565,750	$184	$1,166,671,158	26,370,394	$44
CVC-22		Prevention of cervical cancer through screening with PAP: women 30-49 yrs every 3 years linked with timely treatment of pre-cancerous lesions	$583,174,470	4,002,315	$146	$1,091,493,251	22,516,816	$48
CVC-23		Prevention of cervical cancer through screening with PAP: women 30-49 yrs every 3 years linked with timely treatment of pre-cancerous lesions	$831,867,579	4,554,619	$183	$1,660,368,070	26,290,979	$63
CVC-24		Prevention of cervical cancer through screening with PAP: women 30-49 yrs every 3 years linked with timely treatment of pre-cancerous lesions	$955,726,092	4,714,860	$203	$2,285,793,684	27,447,414	$83
CVC-25		Prevention of cervical cancer through screening with VIA:	$512,420,812	4,085,368	$125	$888,388,618	23,064,846	$39

		women 30-49 yrs every 3 years linked with timely treatment of pre-cancerous lesions						
CVC-26		Prevention of cervical cancer through screening with VIA: women 30-49 yrs every 3 years linked with timely treatment of pre-cancerous lesions	$714,276,297	4,615,409	$155	$1,340,709,421	26,726,375	$50
CVC-27		Prevention of cervical cancer through screening with VIA: women 30-49 yrs every 3 years linked with timely treatment of pre-cancerous lesions	$814,928,544	4,767,951	$171	$1,845,726,367	27,901,962	$66
CRC-1		Basic palliative care for Colorectal Cancer: home-based and hospital care with multi-disciplinary team and access to opiates and essential supportive medicines	$212,692,708	3,128	$67,986	$122,483,474	2,949	$41,540
CRC-2		Basic palliative care for Colorectal Cancer: home-based and hospital care with multi-disciplinary team and access to opiates and essential supportive medicines	$236,193,722	5,006	$47,186	$139,288,883	4,718	$29,525
CRC-3		Basic palliative care for Colorectal Cancer: home-based and hospital care with multi-disciplinary team and access to opiates and essential supportive medicines	$247,944,229	5,944	$41,713	$147,691,587	5,602	$26,363
CRC-4		Diagnosis & treatment of colorectal cancer stages I and II (with surgery, chemotherapy and radiotherapy as needed)	$317,755,979	310,289	$1,024	$238,702,827	233,095	$1,024
CRC-5		Diagnosis & treatment of colorectal cancer stages I and II (with surgery, chemotherapy and radiotherapy as needed)	$357,459,009	633,637	$564	$272,958,560	464,692	$587
CRC-6		Diagnosis & treatment of colorectal cancer stages I and II (with surgery, chemotherapy and radiotherapy as needed)	$377,310,524	870,417	$433	$290,086,427	626,379	$463

BRC-1		Basic palliative care for Breast Cancer: home-based and hospital care with multi-disciplinary team and access to opiates and essential supportive medicines	$241,616,078	12,041	$20,067	$162,516,857	29,868	$5,441
BRC-2		Basic palliative care for Breast Cancer: home-based and hospital care with multi-disciplinary team and access to opiates and essential supportive medicines	$282,471,113	19,265	$14,662	$203,342,296	47,789	$4,255
BRC-3		Basic palliative care for Breast Cancer: home-based and hospital care with multi-disciplinary team and access to opiates and essential supportive medicines	$302,898,631	22,877	$13,240	$223,755,015	56,749	$3,943
BRC-4		Diagnosis & treatment of breast cancer stages I and II (with surgery, radiotherapy and chemotherapy and hormone therapy as needed)	$327,146,264	335,651	$975	$273,470,721	584,274	$468
BRC-5		Diagnosis & treatment of breast cancer stages I and II (with surgery, radiotherapy and chemotherapy and hormone therapy as needed)	$367,425,888	629,010	$584	$324,093,436	1,080,913	$300
BRC-6		Diagnosis & treatment of breast cancer stages I and II (with surgery, radiotherapy and chemotherapy and hormone therapy as needed)	$387,367,687	816,200	$475	$349,098,508	1,389,662	$251
BRC-7		Screening with mammography (once in 2 years for the age group 50 to 69 years) linked with timely diagnosis and treatment	$621,008,808	745,528	$833	$721,995,102	1,237,705	$583
BRC-8		Screening with mammography (once in 2 years for the age group 50 to 69 years) linked with timely diagnosis and treatment	$912,966,894	1,298,852	$703	$1,112,459,442	2,159,801	$515
BRC-9		Screening with mammography (once in 2 years for the age group 50 to 69 years) linked with timely diagnosis and treatment	$1,060,902,271	1,627,782	$652	$1,309,823,117	2,697,617	$486
